# Genome-wide Determinants of Proviral Targeting, Clonal Abundance and Expression in Natural HTLV-1 Infection

**DOI:** 10.1371/journal.ppat.1003271

**Published:** 2013-03-21

**Authors:** Anat Melamed, Daniel J. Laydon, Nicolas A. Gillet, Yuetsu Tanaka, Graham P. Taylor, Charles R. M. Bangham

**Affiliations:** 1 Department of Immunology, Wright-Fleming Institute, Imperial College London, London, United Kingdom; 2 Molecular and Cellular Epigenetics, Interdisciplinary Cluster for Applied Genoproteomics (GIGA) of University of Liège (ULg), Liège, Belgium; 3 Graduate School and Faculty of Medicine, University of the Ryukyus, Okinawa, Japan; 4 Department of Genitourinary Medicine and Communicable Diseases, Wright-Fleming Institute, Imperial College London, London, United Kingdom; Fred Hutchinson Cancer Research Center, United States of America

## Abstract

The regulation of proviral latency is a central problem in retrovirology. We postulate that the genomic integration site of human T lymphotropic virus type 1 (HTLV-1) determines the pattern of expression of the provirus, which in turn determines the abundance and pathogenic potential of infected T cell clones in vivo. We recently developed a high-throughput method for the genome-wide amplification, identification and quantification of proviral integration sites. Here, we used this protocol to test two hypotheses. First, that binding sites for transcription factors and chromatin remodelling factors in the genome flanking the proviral integration site of HTLV-1 are associated with integration targeting, spontaneous proviral expression, and in vivo clonal abundance. Second, that the transcriptional orientation of the HTLV-1 provirus relative to that of the nearest host gene determines spontaneous proviral expression and in vivo clonal abundance. Integration targeting was strongly associated with the presence of a binding site for specific host transcription factors, especially STAT1 and p53. The presence of the chromatin remodelling factors BRG1 and INI1 and certain host transcription factors either upstream or downstream of the provirus was associated respectively with silencing or spontaneous expression of the provirus. Cells expressing HTLV-1 Tax protein were significantly more frequent in clones of low abundance in vivo. We conclude that transcriptional interference and chromatin remodelling are critical determinants of proviral latency in natural HTLV-1 infection.

## Author Summary

HTLV-1 is a human retrovirus, estimated to infect over 10 million individuals worldwide, which causes the inflammatory disease HTLV-1-associated Myelopathy/Tropical Spastic Paraparesis and an aggressive malignancy known as Adult T-cell Leukemia/Lymphoma. The mechanisms that allow the virus to maintain a life-long infection are not fully understood. Here we identified attributes of the host genome flanking the integrated HTLV-1 provirus associated with integration targeting and spontaneous expression of the provirus in vitro, and clonal expansion in vivo. Spontaneous expression (after short-term culture) of the viral protein Tax, which is known to drive proliferation of the infected cell, was significantly more frequent among less expanded clones, suggesting that Tax-expressing clones are more efficiently controlled by the immune response. Certain transcription start sites immediately upstream of the viral integration site were associated with virus latency, which in turn was associated with clonal expansion in vivo.

## Introduction

It is poorly understood how the flanking host genome influences transcription of an integrated provirus. Experiments on artificially modified proviral reporter constructs have yielded contradictory evidence on the role of flanking host promoters in either driving proviral transcription, or suppressing it by transcriptional interference [1], [2]. Conclusions from experiments on single artificial clones therefore cannot be reliably generalized: evidence is required from genome-wide studies of integrated proviruses in natural infection.

Human T lymphotropic virus Type 1 (HTLV-1) persists in vivo by two routes: by driving selective clonal proliferation of infected T lymphocytes (‘mitotic spread’) and by de novo infection (‘infectious spread’) via the virological synapse [3]. HTLV-1 replication is counterbalanced by a strong, chronically activated cytotoxic T lymphocyte (CTL) immune response [4]. The HTLV-1 proviral load (number of proviral copies per 100 PBMCs) varies between infected individuals by over 1000-fold. The proviral load is the strongest correlate of HTLV-1 associated diseases, in particular Adult T-cell Leukemia-Lymphoma (ATLL, [5]) and HTLV-1 Associated Myelopathy/Tropical Spastic Paraparesis (HAM/TSP, [6]).

Mitotic spread of HTLV-1 results in expanded clones of cells that carry the provirus in the same genomic integration site [7]. Infectious spread results in integration of the provirus at a new genomic position. We have recently shown that the majority of naturally infected T-cell clones carry a single proviral copy [8]. Integration of HTLV-1 does not favour specific hotspots, but is more frequent in transcriptionally active areas of the genome [9], [10], [11]. However, the factors that determine integration targeting and the abundance and expression of the HTLV-1 provirus in vivo are unknown. Two HTLV-1 gene products are thought to play a crucial role in viral persistence in vivo. Tax, the transcriptional transactivator of the virus, elicits abundant, chronically activated CTLs [12], [13], [14], indicating continuous or repeated expression of Tax in vivo. Ex vivo, Tax protein is spontaneously expressed in a fraction of infected peripheral blood mononuclear cells (PBMCs) after overnight culture [15]. *HBZ* is the only gene expressed from the minus strand of the provirus. *HBZ* also promotes infected cell proliferation [16] and the CTL response to HBZ protein is a key determinant of proviral load and the risk of the inflammatory disease HAM/TSP [17], [18]. Tax enhances HBZ expression; HBZ protein exerts negative feedback on Tax expression [19], [20].

We hypothesize that the genomic integration site of HTLV-1 determines the pattern and intensity of expression of the plus and minus proviral strands, which in turn determine the equilibrium abundance and the pathogenic potential of an infected T cell clone in vivo. To test this hypothesis, we used our recently described protocol [11] of high-throughput mapping and quantification of proviral integration sites in fresh primary PBMCs from HTLV-1-infected individuals.

## Results

### HTLV-1 preferentially integrates within 1 kb of a host transcription start site and is strongly biased to specific transcription factor binding sites

**Figure 1:**
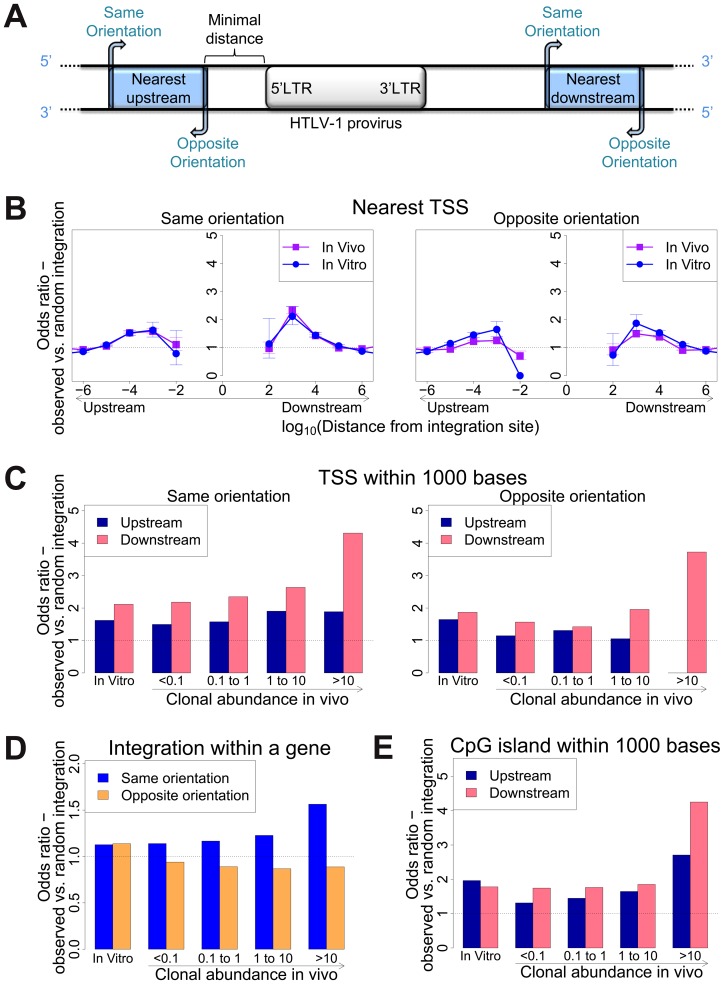
Genomic environment at HTLV-1 proviral integration site determines integration in vitro and abundance in vivo. (A) Blue blocks denote a genomic feature such as a transcription start site. The distance to the nearest genomic feature is calculated (unless otherwise stated) separately for features upstream (closer to 5′ LTR) and downstream of the provirus. Unless otherwise stated, distance is calculated to the nearest end of the genomic feature. Where the genomic feature has an orientation (i.e. transcription units) its orientation relative to the transcriptional orientation of the provirus is indicated as “same” or “opposite”. (B) to (E): proportion of observed integration sites compared to random expectation. (B) Frequency of integration in proximity to transcriptional units (RefSeq). In vitro denotes a combined dataset from two independent experiments (see Table 1). (C) Frequency of integration within 1 kb of a TSS according to clonal abundance (cells in a given clone per 10 000 PBMCs). (D) The excess frequency (compared with random) of observing a provirus within a transcription unit was greater among abundant clones in vivo integrated in the same transcriptional orientation (blue) but not in opposite orientation (orange). (E) The excess frequency (compared with random) of observing a provirus within 1 kb of a host CpG island increased with increasing clonal abundance, in particular where the CpG island lay downstream of the integration site.

To identify genomic factors associated with the targeting of HTLV-1 integration, we infected Jurkat T cells by short co-culture with the HTLV-1-producing cell line MT2. The integration sites were then analysed using our high-throughput protocol and compared to a control list of random sites in the human genome. Figure 1A illustrates the possible orientations (same or opposite) of the nearby genomic features, such as transcription start sites, either upstream or downstream of the integrated provirus.

We previously showed [11] that 47% of de novo HTLV-1 proviral integration events lie within a RefSeq gene. This frequency is slightly higher than expected by chance, but is much lower than that observed for HIV (∼70%), which uses the host protein LEDGF to target proviral integration to genes [21]. As expected by chance, ∼50% of proviruses integrated within host genes were in the same transcriptional orientation as the host gene (Figure 1D, in vitro).

Gillet et al [11] reported a significantly higher than expected proportion of in vitro integration sites within 10 kb of a RefSeq gene. We extended this analysis to identify the optimal (most frequent) distance between the integration site and the nearest host transcription start site (TSS). The results (Figure 1B) show a peak preference (measured by the odds ratio, OR, observed/expected) towards integration in proximity to TSS at ∼1 kb of the integrated provirus (upstream or downstream); the OR gradually diminished until it reached 1 (same as random expectation) at ∼1 Mb from the integration site (Figure 1B). There was a small bias (non-significant for in vitro integration) towards integration with a TSS downstream of the integration site (Figure 1C, in vitro).

Similarly, we observed a bias (up to 2-fold greater than random) towards integration in proximity to CpG islands; again, the bias reached a peak at 1 kb from the nearest CpG island (supplementary Figure S4).

We showed previously [11] that HTLV-1 provirus preferentially integrates in transcriptionally active regions of the host genome. To test the hypothesis that specific transcription factor binding sites (TFBS) influence HTLV-1 proviral targeting, expression and clonal abundance, we used data on genome-wide TFBS ChIP-seq: where available, from primary CD4^+^ T cells; otherwise, from T cells or other human cell types; see Table S3 for complete listing of the datasets used.

**Figure 2:**
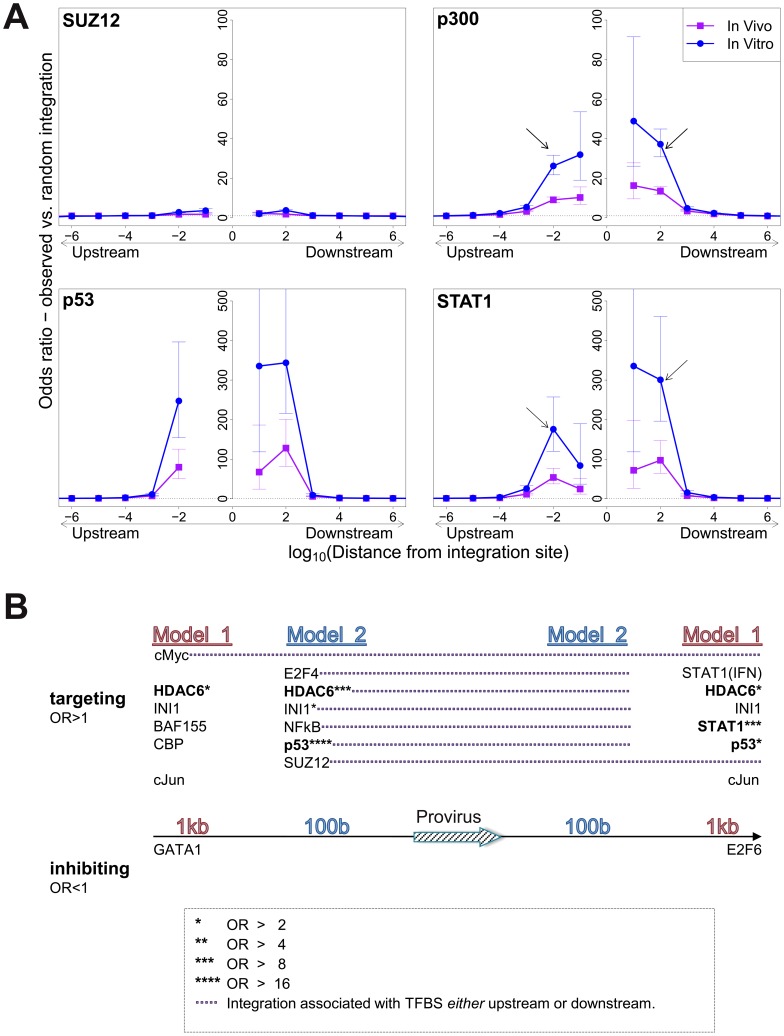
Influence of host TFBS on integration site targeting. (A) Bias in integration in proximity to TFBS (based on ChIP-seq experiments), measured by the odds ratio compared to random expectation. Four representative plots are shown; see also supplementary information. The excess frequency of integration in proximity to TFBS was frequently greater in in vitro infection than in clones isolated from PBMCs in vivo, and greater in low abundance clones in vivo than high abundance clones in vivo (see bottom right panel and supplementary information). Arrows indicate a symmetrical (p300) or asymmetrical (STAT1) bias towards integration in proximity to TFBS, as well as a lower bias in close proximity to IS (STAT1). See also supplementary Table S4 for underlying data. (B) TFBS independently associated with integration frequency in vitro were identified by multivariate analysis. OR – odds ratio. TFBS shown above the line were associated with an excess frequency of integration compared with random (OR>1); TFBS below the line were significantly less likely to lie near the provirus (OR<1). Model 1 and Model 2 (carried out independently) test for TFBS within 1 kb and 100 bp of IS, respectively.

In vitro integration sites showed a remarkably strong bias (compared with random sites) towards integration in proximity to specific TFBS, in particular STAT1, p53, HDACs (e.g. HDAC3, HDAC6) and HATs (e.g. p300, CBP) (Table S3). In most cases the effect was localized to within 100–1000 bases of the integration site (Figure 2A) and declined sharply at greater distances. Two patterns were observed in this biased integration. First, the preference towards integration in proximity to TFBS was typically symmetrical (e.g. p300), i.e. equally strong upstream and downstream of the integration site but in some cases was asymmetrical (e.g. STAT1), with a bias towards one side (often downstream). Second, in many cases we observed a sharp decrease in the preferential integration at 10 bases from the TFBS, such as STAT1 Figure 2A). This pattern was consistently observed across several in vitro and in vivo datasets (supplementary Figures S1, S2).

Because certain TFBS are frequently co-located in the human genome [22], we wished to test which TFBS were independently associated with targeting of the integration site. First, a likelihood ratio test was used to test whether the TFBS was selectively associated with integration either upstream or downstream of the integration site, and each TFBS was then tested individually using a univariate model. We then combined all significant factors using a step-down multivariate logistic regression analysis until only independently significant (p<0.05) factors remained. Most factors that were independently associated with integration site targeting occurred with equal frequency upstream or downstream of the integration site (Figure 2B, see also supplementary Table S7). The factors with the highest odds ratios were the transcription factor p53 and the histone deacetylase HDAC6.

### Effect of HTLV-1 integration sites on clonal expansion

We previously reported [11] a significant association between certain features in the flanking genome and in vivo expansion of the infected T-cell clone. Here, we found that proviruses integrated within a gene were more frequent in larger (more abundant) clones than in smaller clones in vivo, but only when the provirus was integrated in the same transcriptional orientation as the host gene (Figure 1D); the frequency of integration in the opposite orientation was not positively correlated with clonal abundance.

High clone abundance (Figure 1C, top two bins) was associated with the presence of a host TSS within 1 kb downstream of the provirus; here, the transcriptional orientation of the provirus had less effect on abundance than in the case of proviruses integrated within a host gene. The excess frequency of TSS downstream (but not upstream) was much higher in integration sites in vivo than in vitro, in particular when the provirus was integrated in the same orientation as the nearby host gene (p(same) <10^−5^; p(opposite) <0.05, χ^2^ test). The presence of a host CpG island within 1 kb downstream was also selectively associated with clone high abundance (Figure 1E).

**Figure 3:**
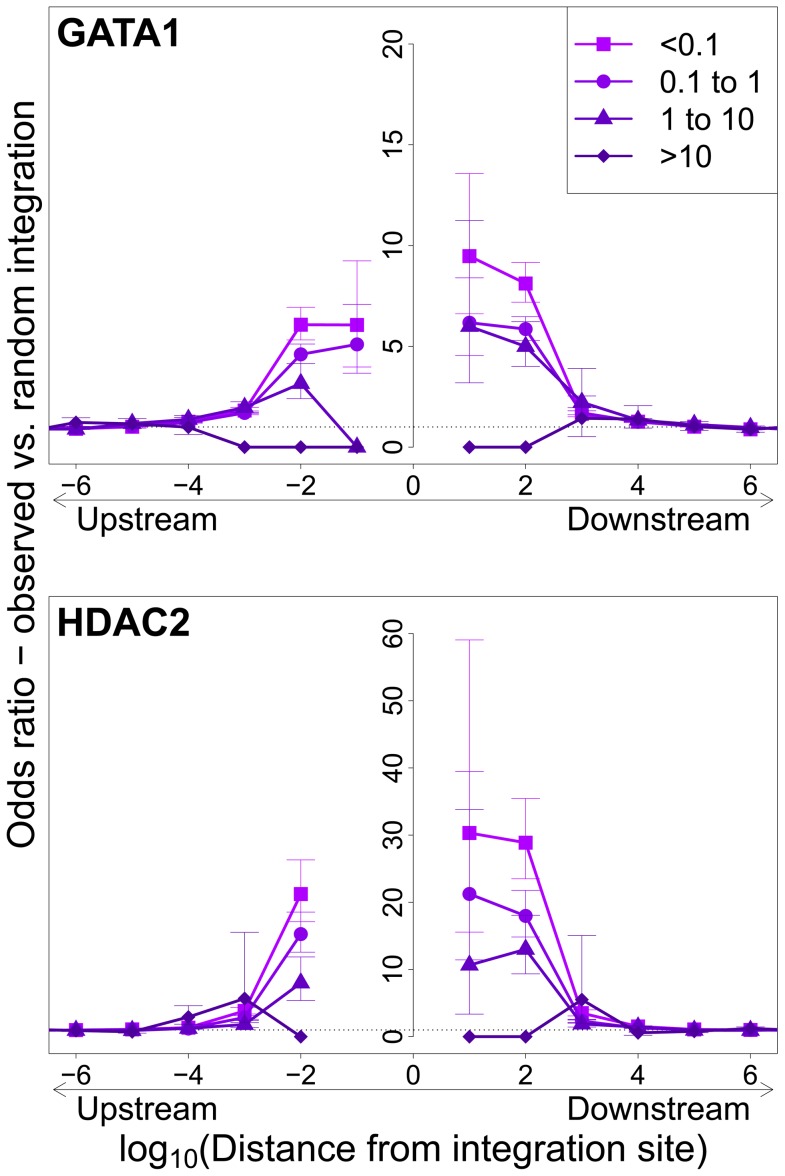
Influence of host TFBS on clonal abundance. Bias in integration in proximity to TFBS (based on ChIP-seq experiments), measured by the odds ratio compared to random expectation. Two representative plots are shown; see also supplementary Figure S3. The excess frequency of integration in proximity to TFBS was greater in low abundance clones in vivo than high abundance clones in vivo. See also supplementary Table S5 for underlying data.

Integration sites observed in vivo showed a similar bias towards proximity to TFBS, with two important differences. First, the OR was in each case lower than that observed in in vitro integration. Second, the magnitude of the bias (OR) declined as clonal abundance increased (Figure 3; supplementary Figure S3).

### Effect of HTLV-1 integration site on Tax expression

We wished to identify features of the genomic integration site that favour expression of the HTLV-1 provirus. We hypothesized that the genomic environment flanking the proviral integration site determines the rate of spontaneous expression of the HTLV-1 transactivator protein Tax by a given infected T-cell clone: that is, the proportion of cells in that clone that express Tax within a given time interval. CD8^+^ T-cells were depleted from fresh unstimulated PBMCs of 10 infected HAM/TSP patients (to preclude CTL-mediated lysis), and the CD8^−^ population was incubated in vitro overnight to allow spontaneous expression of the Tax protein [15]. We then sorted the cells by flow cytometry to isolate Tax^+^ and Tax^−^ cells and analysed the integration sites in the two cell fractions.

**Figure 4:**
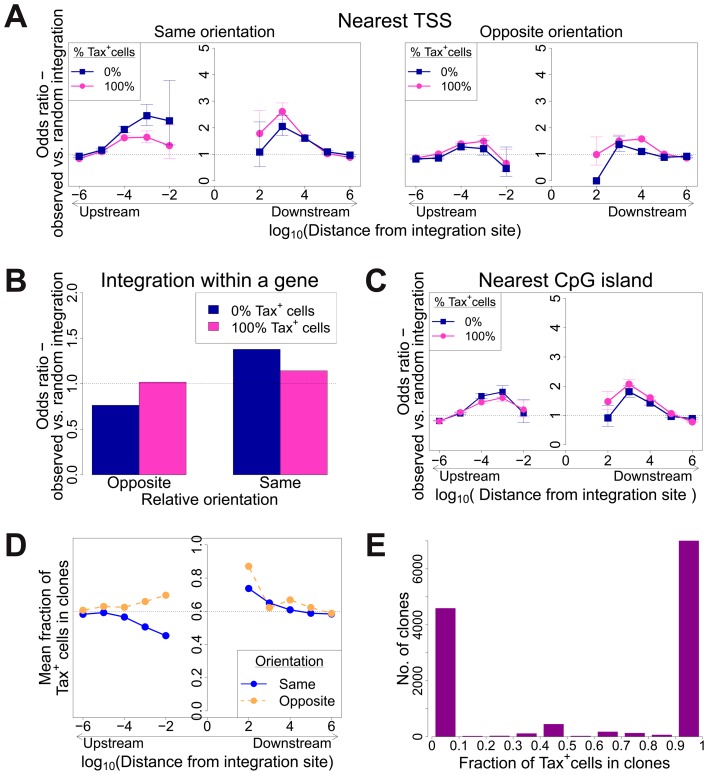
Genomic environment at HTLV-1 proviral integration site associated with proviral expression after 18 h in culture. CD8-depleted PBMCs were placed in culture overnight and sorted by flow cytometry to isolate Tax^+^ and Tax^−^ cells, followed by integration site analysis of sorted cells. (A)–(C): proportion of observed integration sites compared to random expectation. (A) Frequency of integration in proximity to transcriptional units (RefSeq) in clones that were 100% Tax^+^ or 100% Tax^−^, according to the relative transcriptional orientation of the provirus and the host gene. The peak of integration at 1 kb mirrors that observed in vivo in unsorted cells (Figure 1B). However, the integration site in Tax^−^ clones was more likely than in Tax^+^ clones to possess a nearby upstream TSS in the same orientation, and less likely to lie nearby a downstream TSS in the same orientation (or any relative position in the opposite orientation). (B) The provirus in Tax^−^ clones (blue) was oriented in the same transcriptional sense as the host gene in which it was integrated more frequently than random. The orientation of Tax^+^ clones (pink) did not differ from random. (C) Frequency of integration in proximity to CpG islands in clones that were 100% Tax^+^ or 100% Tax^−^. The peak of integration at 1 kb mirrors that observed in vivo in unsorted cells and in vitro (Figure S4). (D) Mean fraction of Tax^+^ cells in clones with a TSS at a given distance (log scale) from the integration site, according to the relative transcriptional orientation of the provirus and the host TSS. The dotted line denotes the mean fraction of Tax^+^ cells across all clones. (E) Frequency distribution of clones according to the frequency of Tax^+^ cells in the respective clones. See supplementary Figure S7 for detailed frequency distribution separated according to clone abundance.

We measured the proportion of each clone that spontaneously expressed Tax by quantifying individual integration sites in the Tax^+^ and Tax^−^ cells, (Figure 4E, and supplementary Figure S7). The observed proportion of Tax^+^ cells per clone varied between 0% and 100%. The majority of clones, regardless of clonal abundance, were either >90% Tax^+^ or >90% Tax^−^. This observation is consistent with the hypothesis that the rate of spontaneous expression of Tax is an intrinsic property of each clone and is determined by the proviral integration site.

When the provirus was integrated within a host gene, we observed a slight but significant excess frequency of Tax^+^ cells compared with Tax^−^ cells (46% vs 43% respectively, p<10^−3^, χ^2^ test). However, while the proviruses in the Tax^+^ cells were found with equal frequency in the same or the opposite transcriptional orientation to the host gene in which they were integrated, the Tax^−^ cells were significantly more frequently present in the same orientation as the host gene (52% of Tax^+^ vs 59% of Tax^−^ cells, p<10^−15^, χ^2^ test). Thus, T cell clones that were 100% Tax^−^ were significantly more likely to carry a provirus in the same orientation as the host gene (Figure 4B).

The relative position (upstream or downstream of the integration site) and the transcriptional orientation of the nearest host gene influenced not only the clonal abundance (Figure 1) but also spontaneous Tax expression. Where the nearest host gene lay in the same transcriptional orientation as the HTLV-1 provirus, the presence of a host TSS (Figure 4A) or CpG island (Figure 4C) within 1 kb upstream of the provirus was associated with silencing of Tax, whereas a TSS or CpG island within 1 kb downstream was associated with Tax expression. The closer the upstream gene was to the integration site, the lower was the proportion of Tax^+^ cells if the gene was in the same orientation (Figure 4D). In contrast, where the nearest host gene was in opposite transcriptional orientation, this asymmetrical effect of the nearby host gene was not observed (Figure 4A, right-hand panel; Figure 4D).

**Figure 5:**
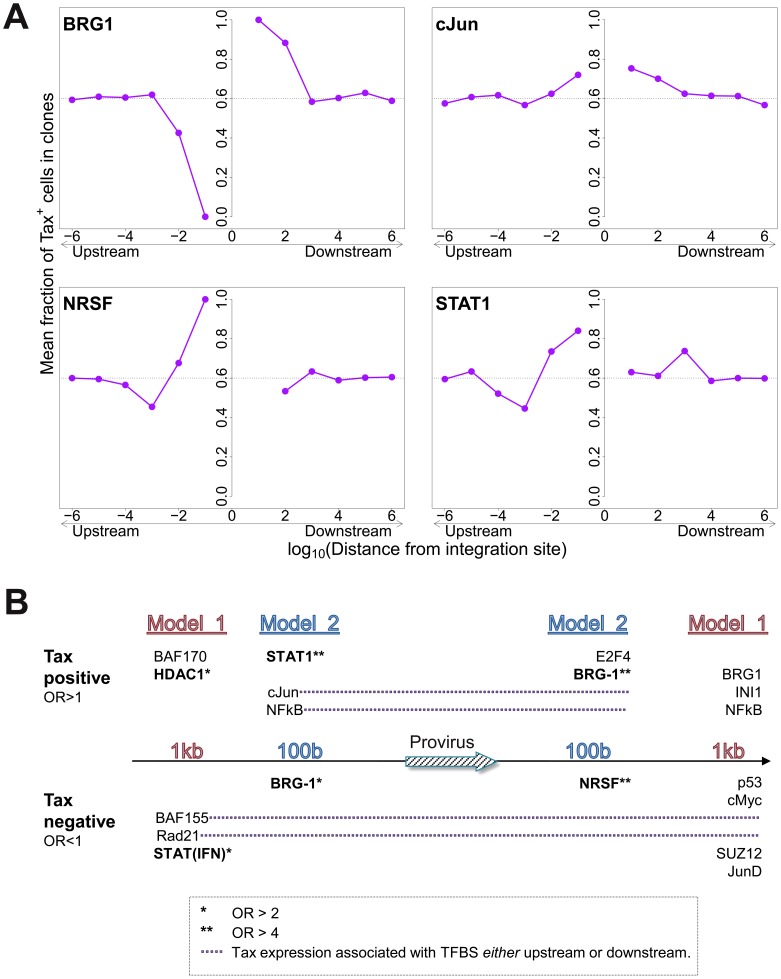
Influence of proximity to TFBS on Tax expression. (A) Mean fraction of Tax^+^ cells in clones with a TFBS (based on ChIP-seq experiments) at a given distance from the IS. Four representative plots are shown. (B) TFBS that were independently associated with Tax expression were identified by multivariate analysis, outcome measure . TFBS shown above the line were associated with Tax expression (OR>1); TFBS below the line were associated with Tax silencing (OR<1). Model 1 and Model 2 (carried out independently) test for TFBS within 1 kb and 100 bp of IS, respectively.

The mean proportion of Tax^+^ cells in one clone (across all clone abundance classes) was 60%. We wished to test whether proximity to TFBS would alter this proportion. We found that the presence of certain TFBS (including STAT1, cJun, NRSF) within 1 kb upstream of the integration site was associated with a higher proportion of Tax^+^ cells in the respective T-cell clone (Figure 5A). A notable exception was BRG-1, which showed a strong opposite asymmetric effect: cells containing a BRG-1 site just upstream of the provirus were more likely to be Tax^−^, whereas cells with a BRG-1 site just downstream of the provirus were more likely to be Tax^+^ (Figure 5A, top left panel).

To identify the TFBS that were independently and significantly associated with spontaneous Tax expression, a logistic regression analysis was carried out as described above (Figure 2B) for integration site targeting. The results (Figure 5B, see also supplementary Table S7) confirmed the asymmetric effects of the BRG-1 binding site, and in addition revealed significant asymmetric associations between Tax expression and several other TFBS, notably STAT1, NRSF, and HDAC1. Thus, a STAT1 binding site 100 bp upstream of the provirus strongly favoured Tax expression, but the presence of a downstream STAT1 binding site was not an independent predictor of Tax expression after multivariate analysis. Conversely, an NRSF binding site 100 bp downstream was a significant predictor of Tax negativity, but the closest upstream NRSF binding site was not independently associated with Tax expression. The asymmetry of these associations contrasts with the predominantly symmetrical associations observed between TFBS and integration site targeting (Figure 2B), and suggests a mechanistic interaction between transcription of the provirus and transcription of the flanking host genome.

### Tax^+^ cells are more frequent in low-abundance clones

To test the hypothesis that the level of Tax expression is correlated with the in vivo abundance of the infected T cell clone, we divided all detected clones into four abundance bins based on the total number of cells observed in each clone. There was a significant negative correlation between clone abundance and the proportion of Tax^+^ cells in the respective abundance bin (Figure 5). That is, small clones were more likely to be Tax^+^, and this likelihood decreased as clone abundance increased. We conclude that, at least in cells from HAM/TSP patients, the majority of spontaneous Tax expression observed is due to the large number of low-abundance clones, rather than a small number of high-abundance clones.

## Discussion

An understanding of the regulation of proviral latency is required for attempts to eradicate latent retroviruses and to optimize retroviral vectors for in vitro and in vivo use. In HIV-1 infection, a reservoir of latently infected cells persists indefinitely in the face of antiretroviral drug therapy and precludes eradication of the infection (reviewed in [23]). In HTLV-1 infection, proviral expression is difficult to detect in fresh PBMCs: however, the strong, chronically activated host immune response and the selective oligoclonal proliferation of HTLV-1-infected T cells argue that the virus is continuously or intermittently expressed in vivo [4], [24].

The abundance of an HTLV-1-infected T cell clone in vivo will be determined by the net effect of two main selection forces: its ability to proliferate and its susceptibility to killing by the strong CTL response [4]. If these forces acted upon all clones equally, the clones would have the same relative abundance in the host. However, Gillet et al [11] showed a wide variation in clone abundance both within and between infected individuals and over time. We hypothesized that this variation between clones is caused by the genomic environment of the integrated provirus, by determining the frequency and intensity of expression of proviral genes, in particular Tax and HBZ, which in turn promote cell proliferation and thereby confer a selective advantage on the infected T cell clone.

To identify the host genomic factors that determine integration site targeting, we mapped and quantified proviral integration sites isolated from two independent in vitro infection experiments. We assume that the pattern of integration observed in short-term in vitro infection reflects the initial pattern of integration in vivo, before the selection exerted during chronic infection. The results confirmed our previous observations [10], [11] that the virus is targeted to transcriptionally active regions of the genome, within or near to a host gene. There was no bias in the orientation of the provirus in the initial infection, indicating that the bias observed in integration sites isolated from PBMCs is a result of the long-term selection forces acting on the infected clones in vivo.

We observed a bias towards integration in proximity to particular transcription factor binding sites. This bias was remarkably strong in certain cases (STAT1, NRSF) in single-factor analysis. Because clusters of different TFBS are frequent in the genome [22], we carried out a multivariate (logistic regression) analysis to identify the TFBS that were independently and significantly associated with an excess frequency of integration. The results (Figure 2B) confirmed the identification of p53, HDAC6 and STAT1 as significant independent correlates of integration. Further independent predictors of integration included Ini1 (see below), cMyc, cJun and NF-kB (Figure 2B). p53 and STAT1 both play important roles in HTLV-1 infection. HTLV-1 dysregulates p53 signalling pathways in vivo [25]; it is not known whether insertional mutagenesis contributes to this dysregulation. HTLV-1 also causes widespread activation of interferon-stimulated genes in vivo, including the key transcriptional regulator STAT1 [25]. A strong association was reported between STAT1 and MLV integration [26]; the authors attributed this to an association between MLV integration and particular epigenetic marks (H3K4me3, H3K4me1 and H3K9ac) at the integration site.

The proportion of all integration sites near any one TFBS was in the minority. This observation indicates that proximity to the transcription factor binding site itself is not sufficient for integration, but suggests that these transcription factors (or an associated host factor) increase the efficiency of proviral integration. Host factors associated with HIV integrase have been thoroughly studied [27]; the most important is the lens epithelium-derived growth factor (LEDGF/p75, [28]), which determines integrase localization [29] and targeting of HIV integrase to transcription units [21]. A study of host factors associated with HTLV-1 integrase is currently underway.

The observed bias towards integration near certain TFBS was predominantly symmetrical and short-range, reaching a maximum at 100b from the integration site and falling to random expectation at ∼10 kb (Figure 2A). In many instances the bias dropped sharply at less than 100b from the integration site: we suggest that this drop is due to steric hindrance between the pre-integration complex and the DNA-bound transcription factor.

In contrast to the symmetry observed in the association between genomic features (such as TFBS) and the frequency of initial integration, we found significant asymmetric interactions between the flanking host genome and the integrated provirus in determining clonal abundance and spontaneous proviral expression. Both the relative position of the nearest host gene (upstream or downstream of the provirus) and its relative transcriptional orientation showed significant associations with clone abundance and expression. Previous studies [1], [2] reported contradictory evidence on the role of an upstream same-sense host promoter in either promoting or suppressing proviral transcription. More recently, Shan et al [30] have shown in Bcl-2-transduced CD4^+^ T cells, infected in vitro with GFP expressing modified HIV, that persistent expression of GFP was associated with opposite sense orientation, while inducible expression was associated with same sense orientation. The evidence obtained here demonstrates that, in natural HTLV-1 infection, the presence of a same-sense host gene promoter upstream of the integrated provirus is associated with inhibition of spontaneous proviral expression, suggesting the operation of transcriptional interference. We conclude that the transcriptional interaction between host and HTLV-1 operates at two levels. First, at a regional level – within 10 kb of the provirus – transcriptional activity of the flanking host genome favours proviral gene expression [10], [11], presumably because of accessibility of the euchromatin to transcription complexes. Second, at a local level – within 100b to 1000b – transcriptional interference by a same-sense host promoter within 1 kb upstream can override the regional effect and inhibit proviral transcription.

Two observations reported here demonstrate that proviral integration and expression are not determined simply by the accessibility of chromatin. First, whereas some TFBS were associated with HTLV-1 proviral integration more frequently than expected, the frequency of other TFBS showed no such bias. Second, the asymmetric associations observed between proviral orientation and position with respect to flanking host genes and the abundance and expression of the HTLV-1 provirus argue for a mechanistic interaction between transcription of the HTLV-1 provirus and transcription of the flanking host genome.

An observation of particular interest is the opposing effect of a BRG-1 binding site upstream and downstream of the provirus (Figure 5A). BRG-1, one of the two ATPase components required for the activity of the SWI/SNF complex [31], controls gene expression by remodelling chromatin, i.e. by repositioning nucleosomes to control the access of transcriptional complexes to the DNA. BRG-1 can cause both gene repression [32] and gene activation [33]; the balance appears to depend on which other subunits are recruited to the SWI/SNF complex [34]. Easley et al [35] found that BRG-1 is required for Tax expression and HTLV-1 replication in vitro, and Rafati et al [36] found that the BAF subclass of the SWI/SNF complex repressed HIV-1 transcription whereas the PBAF subclass promoted transcription. Our observation (Figure 5A) that a BRG-1 site upstream of the provirus is associated with silencing of Tax, while a BRG-1 site downstream is associated with Tax expression, is consistent with our conclusion (above) that transcriptional interference dominates the transcriptional interaction between the provirus and the flanking host genome.

The Ini1 subunit of SWI/SNF interacts directly with HIV-1 integrase [37]; a fraction of Ini1 moves transiently to the cytoplasm to associate with the HIV-1 preintegration complex [38]. However, the function of Ini1 in HIV-1 proviral integration and expression in vivo is not understood. We found that genomic sites for Ini1 binding were significantly associated with HTLV-1 proviral integration (Figure 2B). The presence of an Ini1 site 1 kb downstream of the provirus was associated with spontaneous Tax expression, similar to the effect of the downstream BRG-1 site. Finally, the SWI/SNF subunit BAF155 was overrepresented 1 kb upstream of the integration site (Figure 2B), and was associated with Tax silencing when present either upstream or downstream of the provirus (Figure 5B).

**Figure 6:**
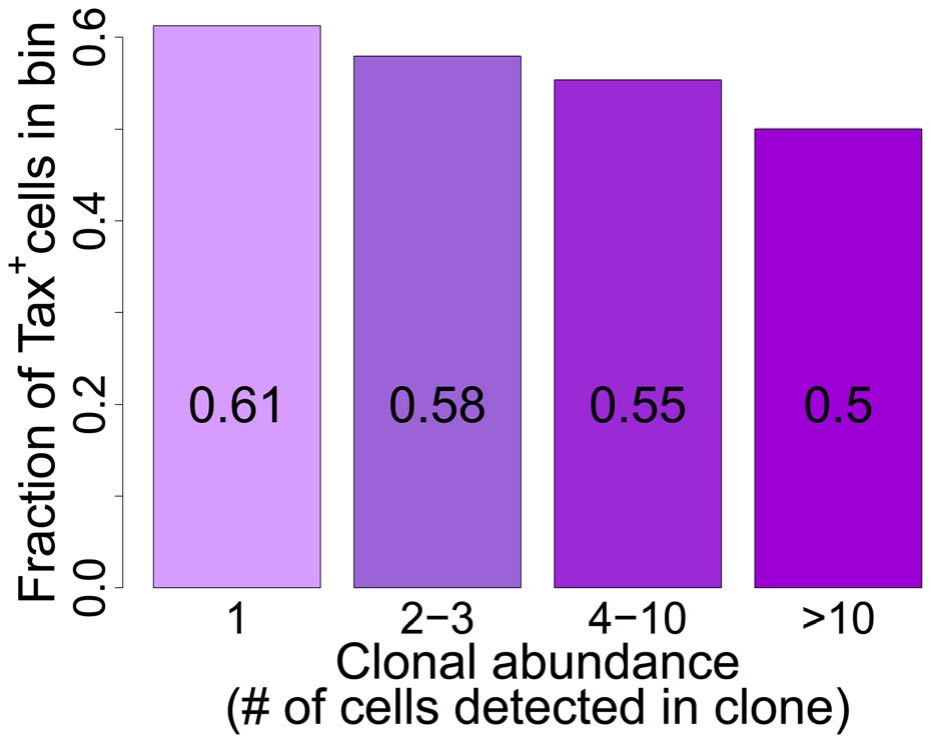
Tax^+^ cells are more frequent in smaller clones. Mean fraction of Tax^+^ cells in bins of increasing clonal abundance (total number of cells in each respective clone). The fraction of Tax^+^ cells was negatively correlated with clonal abundance (p<10^−16^, χ^2^ test for trend).

The HTLV-1 Tax protein acts in concert with host cell transcription factors (notably CBP/p300) on the promoter/enhancer in the viral 5′ LTR, driving plus-strand transcription in a strong positive feedback loop. Tax also acts on response elements for NF-kB, CREB and the serum response factor (SRE) to upregulate expression of a wide range of host genes [39]. Finally, Tax promotes cell cycle progression by accelerating passage through G1 and inhibiting the G1/S and G2/M checkpoints [40]. The net effect of Tax expression is therefore to drive activation and proliferation of the infected T cell. We previously reported that spontaneous Tax expression in fresh unstimulated PBMCs was associated with proliferation of the respective cell in vivo [41]. We therefore expected to observe a positive correlation between the frequency of spontaneous Tax expression by a given clone ex vivo and the abundance of that clone in vivo. However, the results obtained here (Figure 6) demonstrate the opposite, i.e. a highly significant negative correlation. This correlation is likely to be caused by the strong host immune response to the virus. The Tax protein is highly immunodominant in the class 1 MHC-restricted cytotoxic T lymphocyte (CTL) response to HTLV-1 [14], [42], and the *tax* gene is frequently silenced in vivo by mutation or epigenetic changes such as DNA methylation in both untransformed and malignantly transformed (leukemic) cells [43]. Cells that express a high level of Tax are killed by CTLs faster than low Tax-expressing cells [44]. Therefore, suppression or loss of Tax expression may confer a survival advantage on the infected clone in vivo. We conclude that the small (low-abundance) HTLV-1-infected clones express Tax at a higher rate and turn over faster in vivo than the high-abundance clones. It is possible that the critical role of Tax in the HTLV-1 lifecycle is not to maintain clone abundance but rather to promote virion production and infection of new cells by cell contact via the virological synapse [45], [46].

The negative correlation observed between clone abundance and the percentage of Tax^+^ cells, although it was highly significant in all patients combined, was not uniform in every patient. In a small number of patients (in particular those with a high oligoclonality index, Supplementary Figure S8), the most abundant clones (bin 4, clones with greater than 10 cells) contained a high proportion of Tax^+^ cells, suggesting that certain antigen-expressing clones escaped control by the immune response, for example by CTL escape mutations in the *tax* gene [47]. However, clone-specific sequencing of exon 3 of the *tax* gene of 38 clones (>10 cells) from 8 patients did not reveal significant differences in the occurrence of Tax mutations between clones with a high or low frequency of Tax^+^ cells; and only in one patient was a difference in amino acid sequence found between one clone and the others (data not shown).

Interestingly, while Tax^+^ cells were more frequent in low-abundance clones, certain features favouring proviral expression (e.g. a downstream host TSS) also favoured clonal expansion. The association between clonal abundance and proviral integration within 1 kb of a downstream host TSS was maintained even within Tax^−^ clones, consistent with the idea that the selective expansion of these clones is driven by other proviral genes.

These observations raise the possibility that the equilibrium abundance of an HTLV-1-infected T cell clone in vivo is determined not by Tax but by the HBZ gene, encoded on the negative strand of the provirus. Satou et al showed that *HBZ* mRNA promoted proliferation of the infected cell, and whereas Tax expression is frequently undetectable, HBZ appears to be persistently expressed in fresh cells isolated from both non-malignant cases of HTLV-1 infection and cases of adult T-cell leukemia/lymphoma [16]. Further, Macnamara et al recently showed that the CTL response to HBZ is a critical determinant of the equilibrium proviral load in vivo [17]

In this study we examined Tax expression only among CD4^+^ T cells. A small percentage of infected cells are CD8^+^ T cells [48], [49]; it is possible that the genomic factors that determine targeting and expression of HTLV-1 differ in CD8^+^ cells. Also, the propensity of a cell to express Tax was measured by quantifying the frequency of spontaneous Tax expression after 18 hours incubation in vitro. Two lines of evidence suggest that this measure is relevant to HTLV-1 infection and pathogenesis in vivo. First, cells which express Tax ex vivo turn over faster in vivo [41]. Second, the proportion of CD4^+^ cells that express Tax after overnight culture is significantly associated with the HTLV-1 inflammatory disease HAM/TSP [50]. The individuals studied here were all patients with HAM/TSP: the mean level of spontaneous Tax expression is lower in asymptomatic HTLV-1 carriers, but it is unlikely that the molecular mechanisms that govern proviral latency differ qualitatively between asymptomatic carriers and patients with HAM/TSP.

It will be important to compare the present results with the genomic factors associated with HBZ expression or silencing. At present this cannot be done by flow-sorting because existing HBZ-specific antibodies are insufficiently sensitive to detect the low expression levels of HBZ protein in primary cells. We are currently testing the hypothesis that Tax-specific and HBZ-specific CTL clones selectively lyse different clonal populations in vitro.

**Figure 7:**
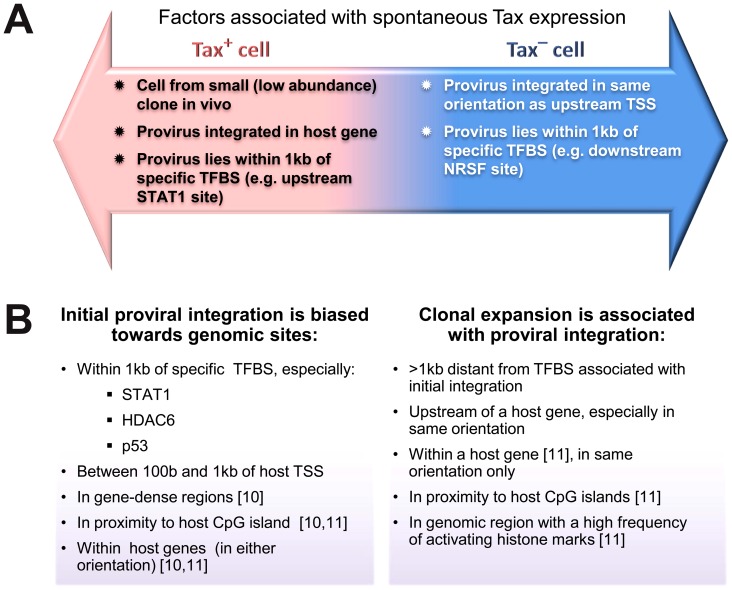
Genomic correlates of HTLV-1 proviral targeting, clonal expansion and proviral expression. (A) Factors associated with the presence or absence of spontaneous Tax expression by a given cell after short-term (18 h) in vitro incubation. (B) Features of the genomic environment of the provirus associated either with initial integration (left panel), or clonal expansion in vivo (right panel). Findings were made in the present study unless otherwise stated. TSS – transcription start site. TFBS – transcription factor binding site.

We have identified host genomic factors that determine the integration site, the proviral expression and selective clonal expansion of HTLV-1 in natural infection in vivo: these factors are summarized in Figure 7. These results open the way to test the molecular mechanisms involved.

## Materials and Methods

### Ethics statement

Blood samples were donated by HTLV-1-infected individuals attending the HTLV-1 clinic at the National Centre for Human Retrovirology (Imperial College Healthcare NHS trust) at St Mary's Hospital, London UK, with fully informed written consent. This study was approved by the UK National Research Ethics Service (NRES reference 09/H0606/106).

### DNA samples (Table 1)

**Table 1:** IS datasets used.

Dataset	total IS	total infected individuals	reference
in vitro (1)	4521	N/A	[11]
in vitro (2)	1805	N/A	This publication
In vivo (1)	78563^1^	63	[11]
In vivo (2)	20202	10	This publication
Tax Negative	6700	10 (pooled)	This publication
Tax Positive	13054	10 (pooled)	This publication
Random UIS	176505	N/A	This publication

1 For the purpose of this work, only one time point was used for each patient (most recent available if multiple time points were originally analysed).

PBMCs were isolated using Histopaque-1077 (Sigma-Aldrich) and cryopreserved in FBS (Gibco) containing 10% DMSO (Sigma-Aldrich). DNA extraction was carried out using the DNeasy Blood & Tissue kit (Qiagen) according to the manufacturer's protocol.

### In vitro infection

In vitro infection was carried out in two independent assays as previously described [11]. Jurkat (JKT) cells were co-cultured for 3 h with γ-irradiated (^137^Cs, 40,000 cGy) MT2 cells [51], labelled with anti-CD4 MicroBeads (Miltenyi). MT2 cells were then depleted from the co-culture using magnetic separation (Miltenyi), and infected JKT cells were maintained in culture for 14 days in RPMI (supplemented by L-glutamine, penicillin, streptomycin) containing 10% FBS for 18 hours at 37C with 5% CO_2_. Genomic DNA was extracted and the proviral integration sites (IS) analysed as previously described [11]. IS from MT2 were also analysed to exclude possible contamination of the JKT IS. No contaminating MT2 IS were found after 14 days.

### Tax sorting

See also supplementary Figure S5, supplementary Table S6. PBMCs from 10 patients with HAM/TSP with a high proviral load (range 12.2–50.6 copies per 100 PBMC) were depleted of CD8^+^ cells using magnetic depletion (Miltenyi) and incubated in RPMI (supplemented by L-glutamine, penicillin, streptomycin) containing 10% FBS for 18 hours at 37C with 5% CO_2_. After 18 h culture, the cells were stained for intracellular expression of Tax (anti-Tax mAb LT4) and sorted using FACS (FACSAria IIIU, BD Biosciences) to isolate two populations of live CD4^+^ cells based on Tax expression. Gates were set (FACSDiva, BD Biosciences) to ensure a clear demarcation between the Tax^+^ and Tax^−^ populations (Figure S6). DNA was extracted from whole unsorted PBMCs from each patient and analysed separately to identify the patient of origin of each clone; 46% of the clones were attributed in this way. To calculate the fraction of Tax^+^ cells in a given clone, the frequency of Tax^+^ and Tax^−^ cells were normalized to the mass of genomic DNA per cell from each respective cell population, to correct for experimental variation in efficiency of genomic DNA isolation (Table S6),

### Analysis of IS

Identification and quantification of proviral integration sites was done as previously described [11]. HTLV-1 infected DNA was randomly sheared by sonication (Covaris S2) and then blunt-ended (Klenow polymerase) and ligated to a partly double-stranded DNA linker. Following a nested PCR step, the resulting DNA libraries were deep sequenced using the Illumina GA-II platform. DNA sequence was aligned to the human genome reference (UCSC hg18, excluding haplotype and “random” sequences) using the ELAND algorithm. Distinct IS were grouped based on integration site and quantified based on number of distinct shear sites isolated and the respective patient's proviral load.

DNA sequences from ∼190000 random sites in the human genome (hg18) were generated using Galaxy [52], [53], [54] and back-aligned to the human genome using the same pipeline to eliminate any potential bias due to alignment limitations.

### Calculation of clonal abundance

The absolute abundance of a given clone was defined as the number of proviral copies of that clone per 10^4^ PBMCs. Given n_i_ - the number of proviral copies for the i^th^ clone, and S – the total number of clones identified in the sample, the absolute abundance was calculated for PBMC samples according to the following formula:
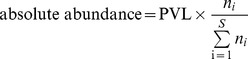
Clone abundance bins were defined on a logarithmic scale since proviral load (used in calculation of abundance) follows a logarithmic distribution [55]. The number of clones in each clone abundance bin is given in Table S1. For samples sorted for Tax protein expression, where proviral load data were not available, the clonal abundance bins were set according to proviral copy count.

### Bioinformatic analysis of genomic environment

Transcription units and CpG island data were retrieved from the NCBI (ftp.ncbi.nih.gov/gene/) and UCSC tables [56], respectively. Annotations to the human genome were obtained from published datasets (Table S3) including ChIP-seq experiments on primary CD4^+^ T cells where available; otherwise, data on human CD4^+^ T cell lines or other human cell lines were used. We used the SISSRs algorithm [57] to identify the position of a putative transcription factor binding site in published ChIP-seq data where raw ChIP-seq data were available.

Annotations positions were compared to the IS using the R package hiAnnotator (http://malnirav.github.com/hiAnnotator), kindly provided by N. Malani and F. Bushman (University of Pennsylvania, USA).

### Statistical analysis

Statistical analysis was carried out using R version 2.13.0 (http://www.R-project.org/). Two separate logistic regression analyses were carried out, respectively, to identify independent predictors of HTLV-1 integration targeting and independent predictors of Tax positivity. Genomic annotations used to derive input variable were published ChIP-seq datasets (see Bioinformatic analysis above; Table S3). For integration targeting, the binary outcome measure was a “true” integration site (from 4521 identified in vitro integration sites) or a “false” integration site (45210 random genomic locations). For spontaneous Tax expression, the binary outcome was Tax positivity (20813 Tax^+^ cells) or Tax negativity (10326 Tax^−^ cells). Each TFBS was tested (presence or absence of the TFBS within a given distance of the integration site) as an independent predictor in each analysis. For each outcome variable, two separate analyses were carried out, respectively at two distances of the integration site - 100 bases and 1 kb.

First, for each TFBS and at each distance, we tested whether the relative position (upstream/downstream) of the integration site and the TFBS determined the outcome by using a likelihood ratio test to compare two competing models: 1) presence or absence of TFBS upstream or (separately) downstream; 2) presence or absence of TFBS, regardless of relative position. Next, we carried out univariate analysis of each individual TFBS, based on the model chosen by the likelihood ratio test. Only TFBS that were significant (p-value<0.05) after correction for multiple comparisons (Benjamini-Hochberg) were used in the multivariate analysis. Multivariate analysis was carried out using a step-down logistic regression method.

## Supporting Information

Figure S1 **Influence of host TFBS on integration site targeting – in vitro.** Bias in integration in proximity to TFBS (based on ChIP-seq experiments), measured by the odds ratio compared to random expectation. The bias was maintained across separate datasets, generated by independent in vitro experiments. Dotted line denotes random expectation (OR = 1).
(TIF)

Figure S2 **Influence of host TFBS on integration site targeting – in vivo.** Bias in integration in proximity to TFBS (based on ChIP-seq experiments), measured by the odds ratio compared to random expectation. The pattern of bias was maintained between different patient clinical groups. Dotted line denotes random expectation (OR = 1). ATLL = Adult T-cell leukaemia/lymphoma . HAM/TSP = HTLV-1 associated myelopathy/Tropical spastic paraparesis. AC = Asymptomatic carrier.
(TIF)

Figure S3 **Influence of host TFBS on clonal abundance in vivo.** Bias in frequency of integration in proximity to TFBS (based on ChIP-seq experiments), measured by the odds ratio compared to random expectation. TFBS that were associated with integration targeting showed a stronger bias (higher OR) in the clones least expanded in vivo. Clonal abundance is expressed as the number of cells in given clone per 10^4^ PBMCs. Dotted line denotes random expectation (OR = 1).
(TIF)

Figure S4 **The genomic environment at the HTLV-1 proviral integration site determines integration targeting in vitro and clonal abundance in vivo.** Frequency of integration in proximity to CpG islands in clones for in vitro (in blue) and in vivo (purple) integration.
(TIF)

Figure S5 **Protocol for flow-sorting of Tax-expressing cells.** (A) CD8^+^ cell-depleted PBMCs were studied from 10 patients with HAM/TSP with a high proviral load. The cells were incubated overnight, fixed and stained for Tax and surface CD4 expression, and sorted on a high-speed flow cytometer (see Figure S6 for details). (B) Recovered cells from all 10 patients were combined in two pools, respectively CD4^+^Tax^+^ cells and CD4^+^Tax^−^ cells. (C) Genomic DNA was extracted from each pool of cells and integration site analysis carried out as described.
(TIF)

Figure S6 **Flow cytometry sorting by Tax expression.** (A) Representative FACS plots of the gating procedure used (from 1 of 10 samples studied). Lower middle panel shows gating of CD4^+^Tax^+^ (‘Tax pos’) and CD4^+^Tax^−^ (‘Tax neg’) populations; these gates were set to distinguish unequivocally between Tax^+^ and Tax^−^ populations. (B) Purity testing of Tax^−^ sorted cells: Tax^+^ cells not detected. C) Purity testing of Tax^+^ sorted cells: 0.2% were Tax^−^.
(TIF)

Figure S7 **Majority of HTLV-1-infected clones were either 100% Tax^+^ or 0% Tax^+^.** Frequency distribution of clones according to the frequency of Tax^+^ cells in each respective clone, binned according to number of sister cells detected in sample: bin 1: 1 cell detected; bin 2: 2 or 3 cells detected; bin 3: 4 to 10 cells detected; bin 4: over 10 cells detected.
(TIF)

Figure S8 **Tax^+^ cells were more frequent in smaller clones.** Mean fraction of Tax^+^ cells within each bin, in bins of increasing clonal abundance (total number of cells in each respective clone). In the majority of patients there was an inverse correlation between clone abundance bin and fraction of Tax^+^ cells: this correlation was highly significant in all patients combined (P<10^−32^). However in certain patients (particularly those with a high oligoclonality Index) the most abundant clones contained a high proportion of Tax^+^ cells. Clone abundance bins were defined as in Figure S7.
(TIF)

Table S1 **In vivo integration sites – sample data by clone abundance.**
(DOC)

Table S2 **In vivo integration sites – sample data by patient code.**
(DOC)

Table S3 **List of annotations datasets used.**
(DOC)

Table S4 **Odds ratio and clone counts data for a selection of TFBS – in vitro, in vivo vs. random sites.**
(XLS)

Table S5 **Odds ratio and clone counts data for a selection of TFBS – clonal abundance bins vs. random sites.**
(XLS)

Table S6 **Tax sorting experiment – sample data.**
(DOC)

Table S7 **Multivariate analysis results – detailed odds ratios and confidence intervals.**
(XLS)
