# Supplementary figures and images for: Genome-wide Determinants of Proviral Targeting, Clonal Abundance and Expression in Natural HTLV-1 Infection
Source: PLoS Pathog. 2013 Mar 21;9(3):e1003271. doi: 10.1371/journal.ppat.1003271 (PMC3605240; doi:10.1371/journal.ppat.1003271)

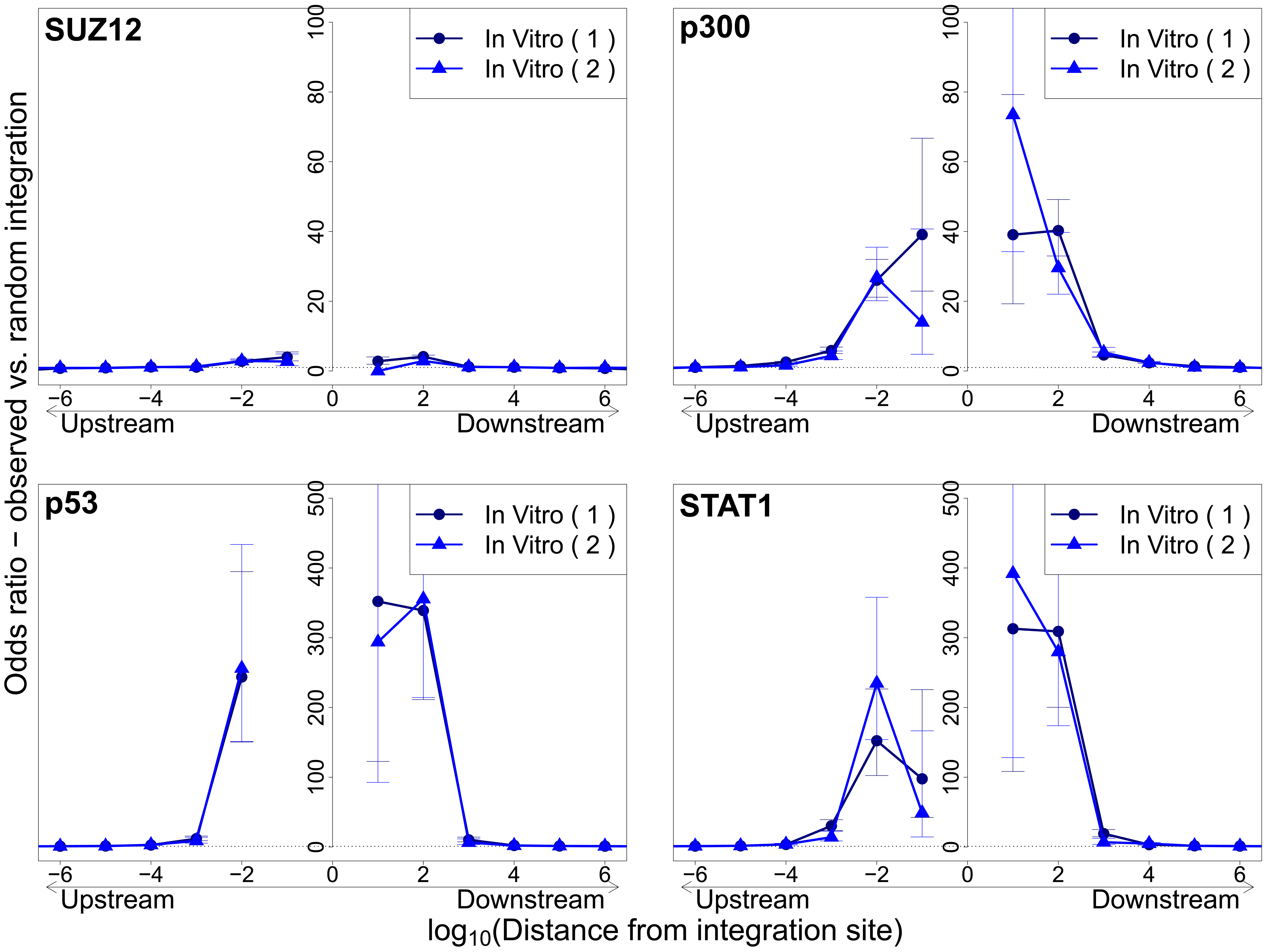

Supplement: Figure S1 — Influence of host TFBS on integration site targeting – in vitro. Bias in integration in proximity to TFBS (based on ChIP-seq experiments), measured by the odds ratio compared to random expectation. The bias was maintained across separate datasets, generated by independent in vitro experiments. Dotted line denotes random expectation (OR = 1). (TIF) [file ppat.1003271.s001.tif]

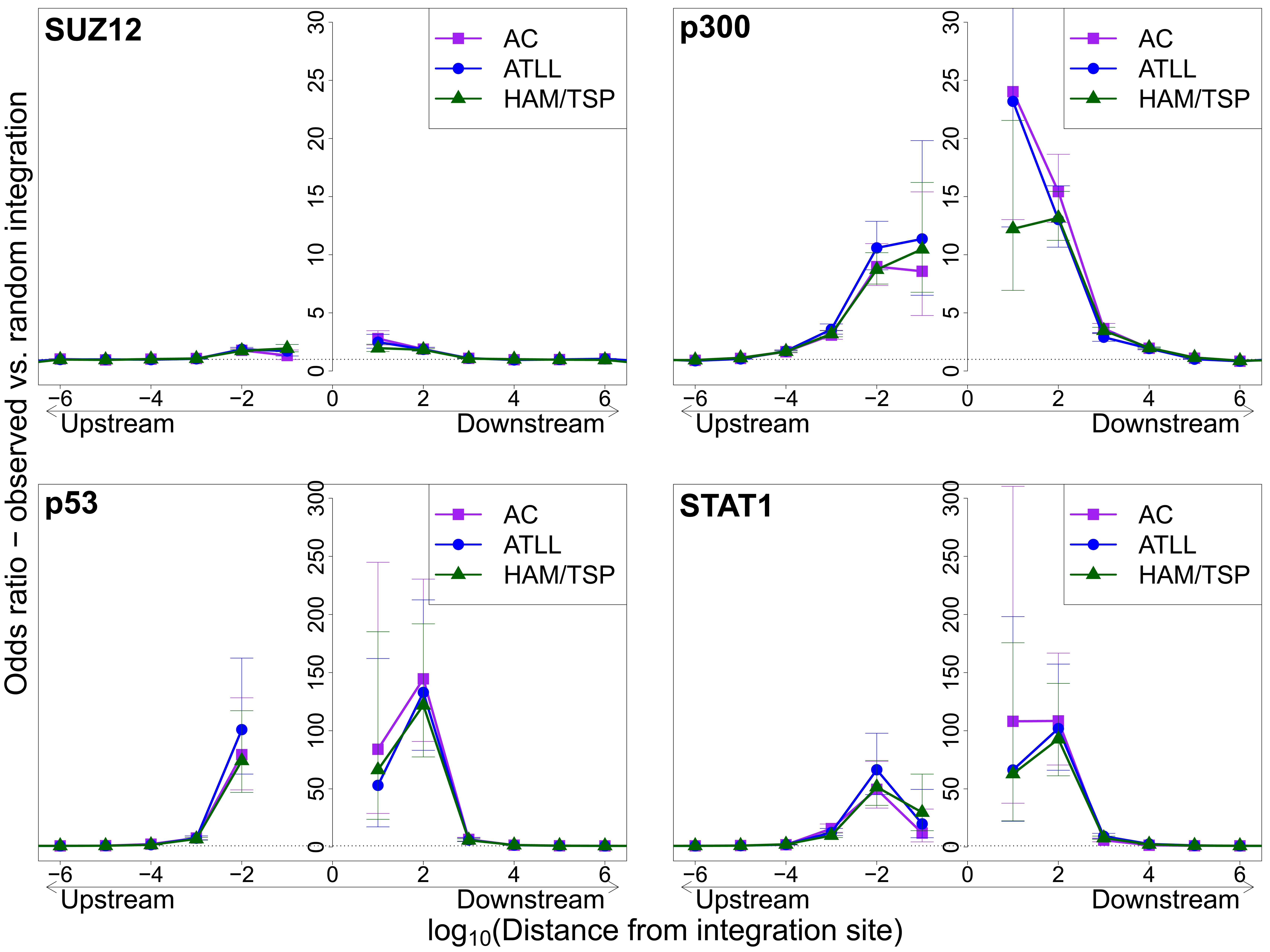

Supplement: Figure S2 — Influence of host TFBS on integration site targeting – in vivo. Bias in integration in proximity to TFBS (based on ChIP-seq experiments), measured by the odds ratio compared to random expectation. The pattern of bias was maintained between different patient clinical groups. Dotted line denotes random expectation (OR = 1). ATLL = Adult T-cell leukaemia/lymphoma . HAM/TSP = HTLV-1 associated myelopathy/Tropical spastic paraparesis. AC = Asymptomatic carrier. (TIF) [file ppat.1003271.s002.tif]

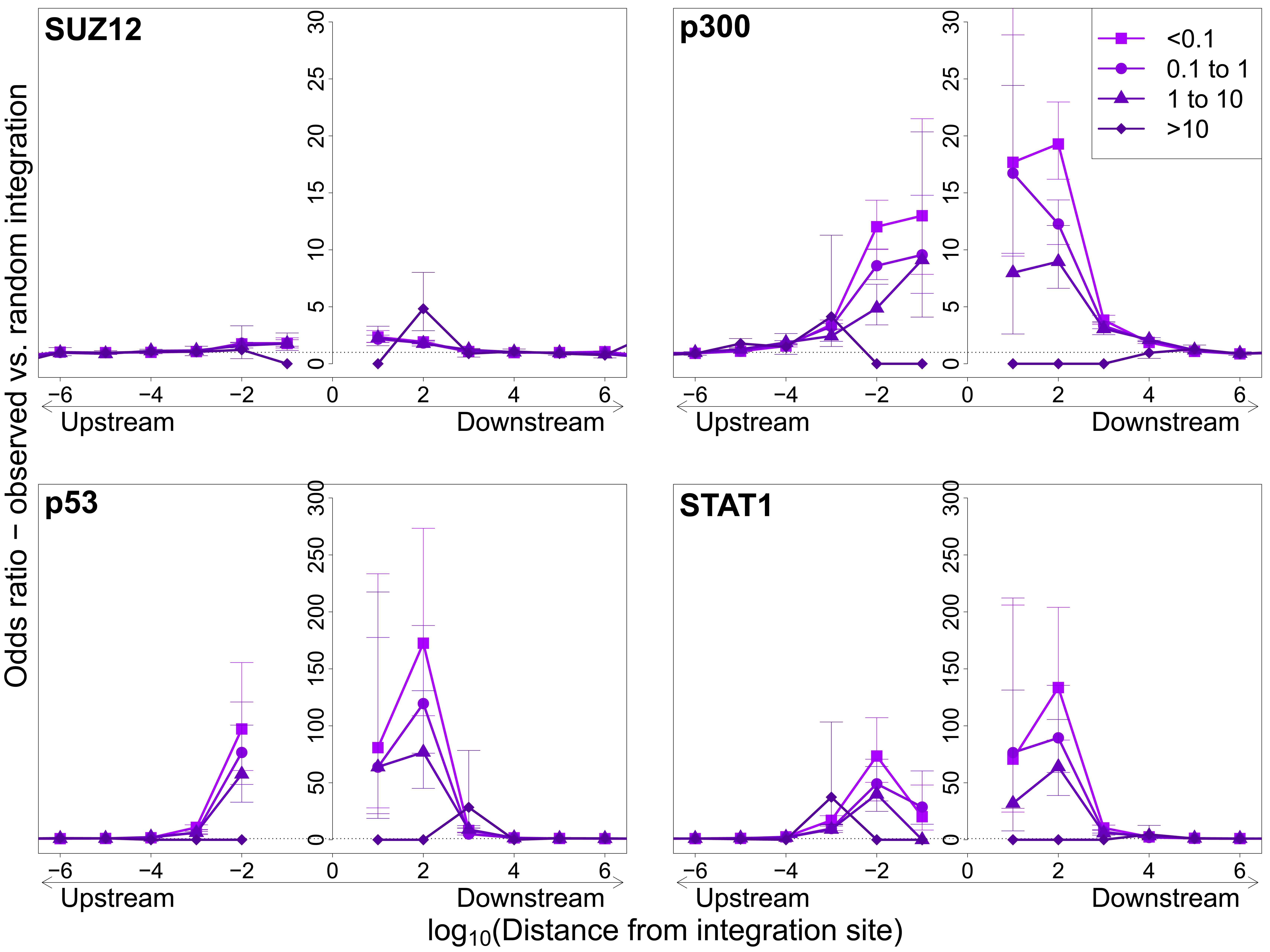

Supplement: Figure S3 — Influence of host TFBS on clonal abundance in vivo. Bias in frequency of integration in proximity to TFBS (based on ChIP-seq experiments), measured by the odds ratio compared to random expectation. TFBS that were associated with integration targeting showed a stronger bias (higher OR) in the clones least expanded in vivo. Clonal abundance is expressed as the number of cells in given clone per 104 PBMCs. Dotted line denotes random expectation (OR = 1). (TIF) [file ppat.1003271.s003.tif]

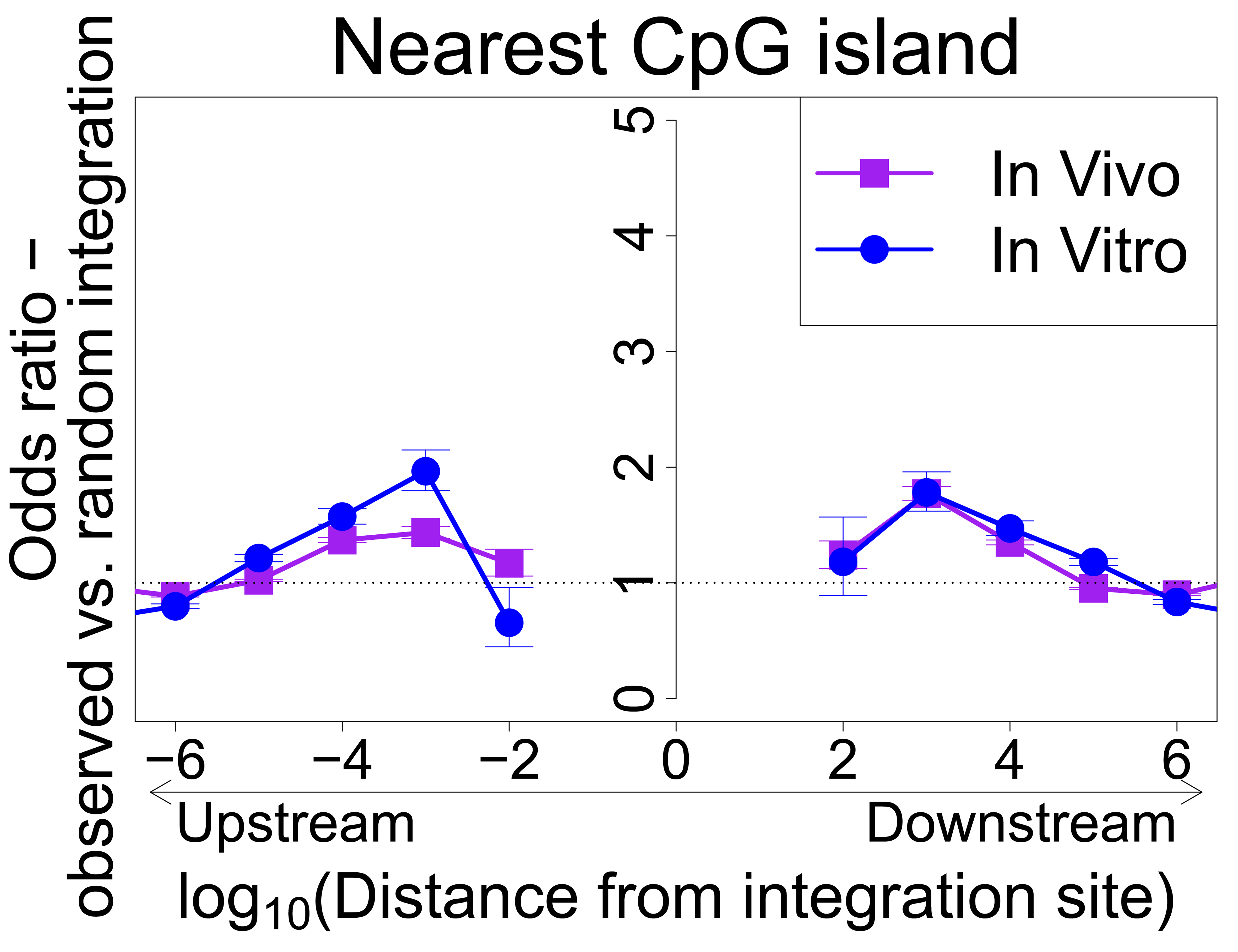

Supplement: Figure S4 — The genomic environment at the HTLV-1 proviral integration site determines integration targeting in vitro and clonal abundance in vivo. Frequency of integration in proximity to CpG islands in clones for in vitro (in blue) and in vivo (purple) integration. (TIF) [file ppat.1003271.s004.tif]

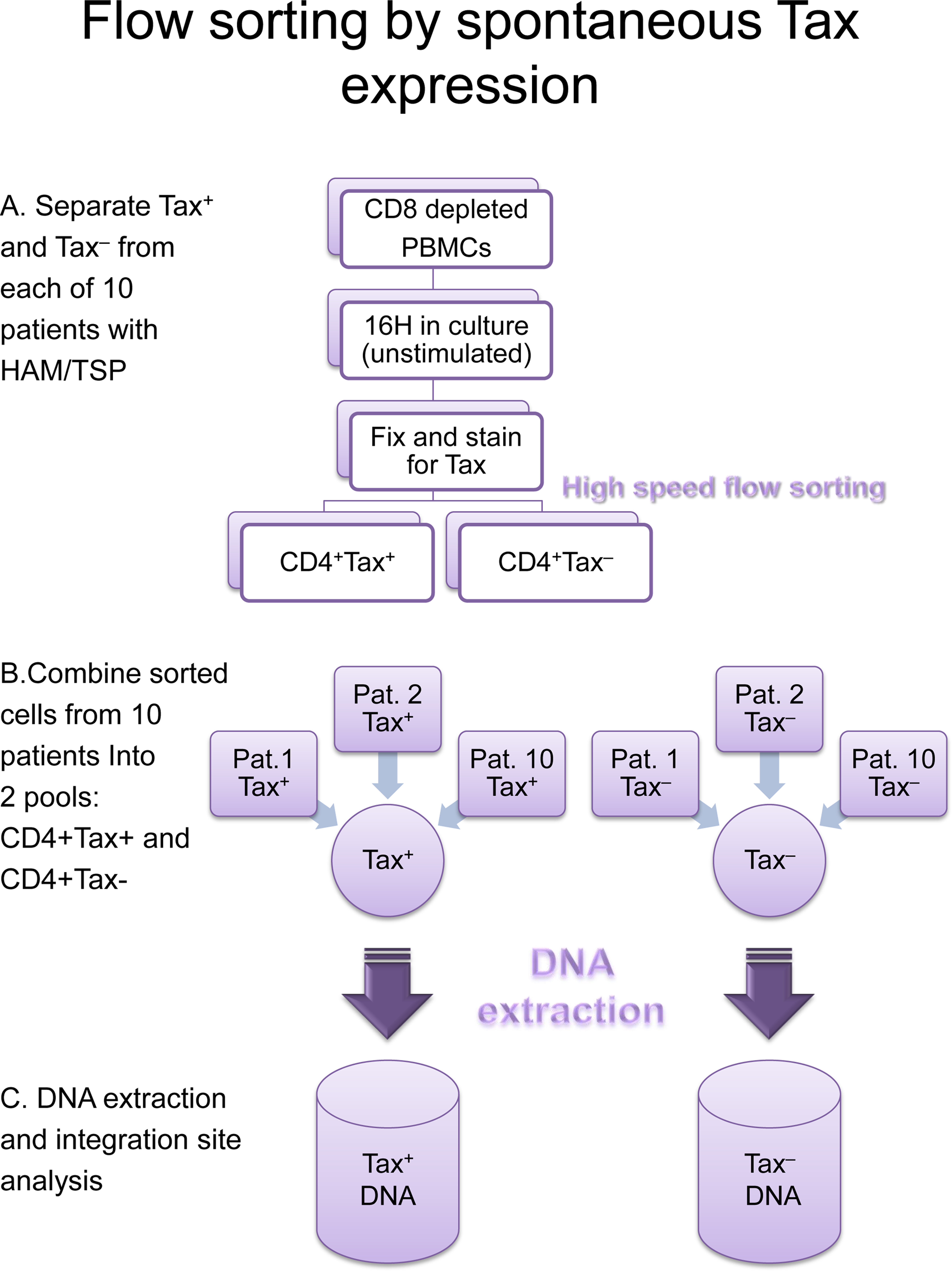

Supplement: Figure S5 — Protocol for flow-sorting of Tax-expressing cells. (A) CD8+ cell-depleted PBMCs were studied from 10 patients with HAM/TSP with a high proviral load. The cells were incubated overnight, fixed and stained for Tax and surface CD4 expression, and sorted on a high-speed flow cytometer (see Figure S6 for details). (B) Recovered cells from all 10 patients were combined in two pools, respectively CD4+Tax+ cells and CD4+Tax− cells. (C) Genomic DNA was extracted from each pool of cells and integration site analysis carried out as described. (TIF) [file ppat.1003271.s005.tif]

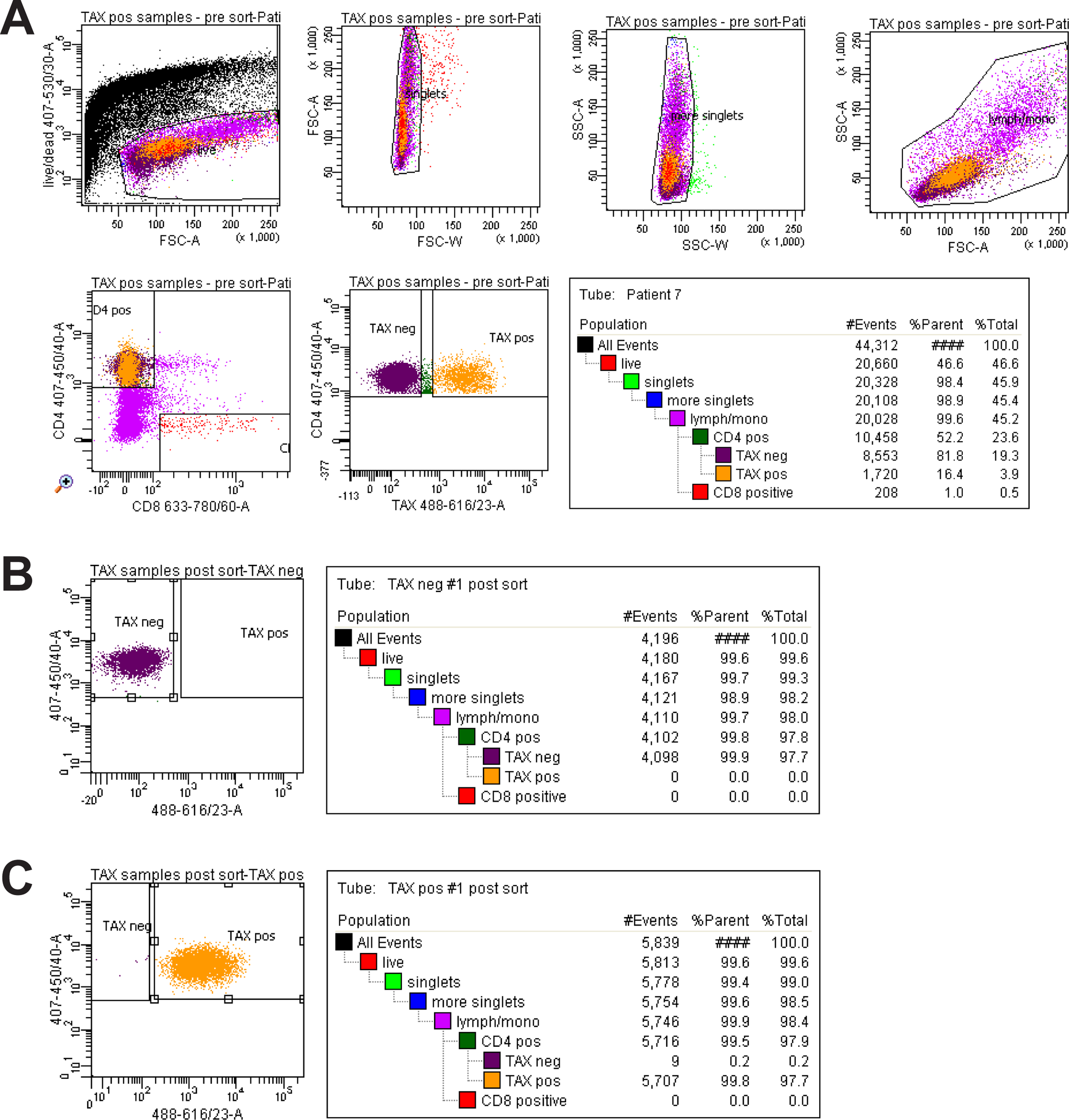

Supplement: Figure S6 — Flow cytometry sorting by Tax expression. (A) Representative FACS plots of the gating procedure used (from 1 of 10 samples studied). Lower middle panel shows gating of CD4+Tax+ (‘Tax pos’) and CD4+Tax− (‘Tax neg’) populations; these gates were set to distinguish unequivocally between Tax+ and Tax− populations. (B) Purity testing of Tax− sorted cells: Tax+ cells not detected. C) Purity testing of Tax+ sorted cells: 0.2% were Tax−. (TIF) [file ppat.1003271.s006.tif]

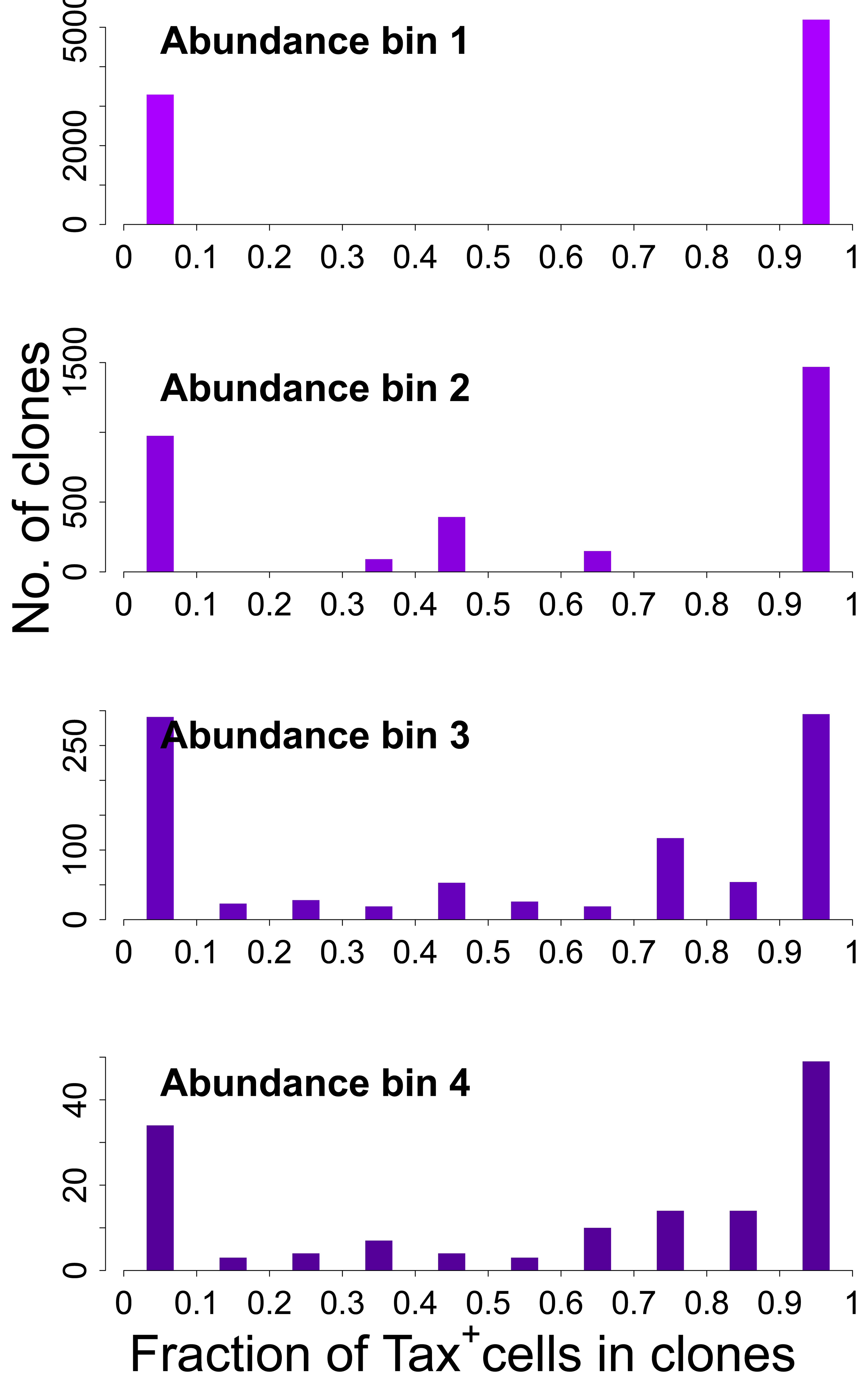

Supplement: Figure S7 — Majority of HTLV-1-infected clones were either 100% Tax+ or 0% Tax+. Frequency distribution of clones according to the frequency of Tax+ cells in each respective clone, binned according to number of sister cells detected in sample: bin 1: 1 cell detected; bin 2: 2 or 3 cells detected; bin 3: 4 to 10 cells detected; bin 4: over 10 cells detected. (TIF) [file ppat.1003271.s007.tif]

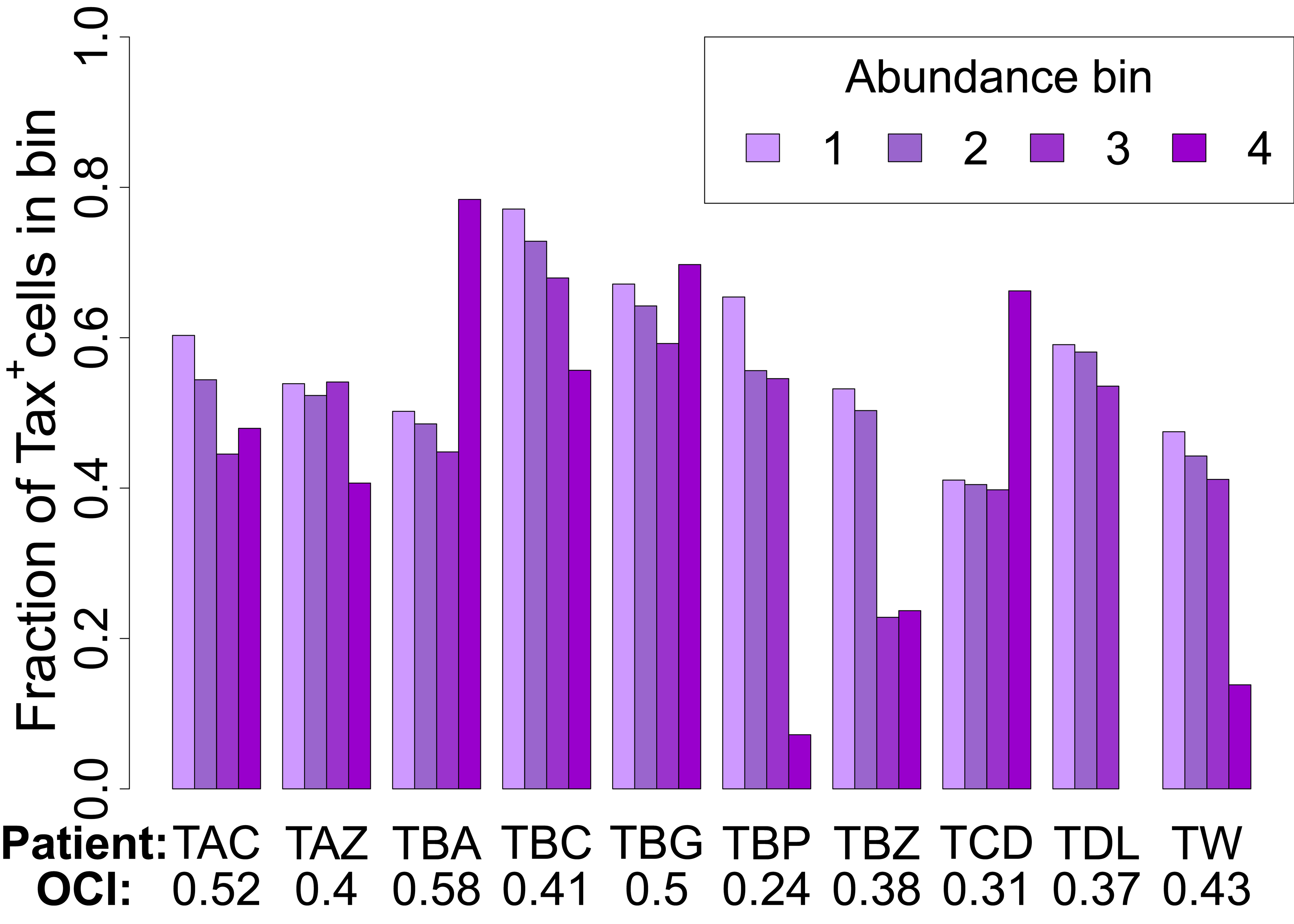

Supplement: Figure S8 — Tax+ cells were more frequent in smaller clones. Mean fraction of Tax+ cells within each bin, in bins of increasing clonal abundance (total number of cells in each respective clone). In the majority of patients there was an inverse correlation between clone abundance bin and fraction of Tax+ cells: this correlation was highly significant in all patients combined (P<10−32). However in certain patients (particularly those with a high oligoclonality Index) the most abundant clones contained a high proportion of Tax+ cells. Clone abundance bins were defined as in Figure S7. (TIF) [file ppat.1003271.s008.tif]
