# Supplementary material for: Genome-wide Determinants of Proviral Targeting, Clonal Abundance and Expression in Natural HTLV-1 Infection
Source: PLoS Pathog. 2013 Mar 21;9(3):e1003271. doi: 10.1371/journal.ppat.1003271 (PMC3605240; doi:10.1371/journal.ppat.1003271)
Supplement: Table S1 — In vivo integration sites – sample data by clone abundance. (DOC) [file ppat.1003271.s009.doc]

**Table S1: In vivo integration sites [1] – sample data by clone abundance**

|  | | **Clone numbers by absolute abundance bin  (proviruses per 10000 PBMC)** | | | | |
| --- | --- | --- | --- | --- | --- | --- |
| **Clinical Status** | **No. Patients** | **<0.1** | **0.1-1** | **1-10** | **>10** | **Total** |
| **AC** | **18** | 3201 | 10275 | 1192 | 32 | **14700** |
| **ATLL** | **20** | 42 | 11627 | 1587 | 67 | **13323** |
| **HAM/TSP** | **25** | 14234 | 33521 | 2748 | 37 | **50540** |
| **Grand Total** | **63** | **17477** | **55423** | **5527** | **136** | **78563** |

AC: Asymptomatic Carriers; ATLL: Adult T-cell Leukaemia/Lymphoma; HAM/TSP: HTLV-1-Associated Myelopathy / Tropical Spastic Paraparesis.

**Additional references:**

1. Gillet NA, Malani N, Melamed A, Gormley N, Carter R, et al. (2011) The host genomic environment of the provirus determines the abundance of HTLV-1-infected T-cell clones. Blood 117: 3113-3122.
