# Supplementary material for: Genome-wide Determinants of Proviral Targeting, Clonal Abundance and Expression in Natural HTLV-1 Infection
Source: PLoS Pathog. 2013 Mar 21;9(3):e1003271. doi: 10.1371/journal.ppat.1003271 (PMC3605240; doi:10.1371/journal.ppat.1003271)
Supplement: Table S2 — In vivo integration sites – sample data by patient code. (DOC) [file ppat.1003271.s010.doc]

**Table S2: In vivo integration sites – sample data by patient [1]**

| Clinical Status | Patient code | Gender | PVL | Sequencing results | | |
| --- | --- | --- | --- | --- | --- | --- |
|  |  |  |  | # sequencing reads | # proviruses | # clones |
| AC | HAY | F | 3.6 | 88587 | 8034 | 2070 |
| AC | HBE | F | 3.6 | 7860 | 1400 | 888 |
| AC | HBF | F | 7.6 | 264490 | 3536 | 1615 |
| AC | HBK | F | 8.9 | 232565 | 4284 | 980 |
| AC | HBT | F | 0.5 | 12183 | 295 | 126 |
| AC | HBX | F | 2.7 | 42889 | 1607 | 642 |
| AC | HBY | M | 1.6 | 10377 | 540 | 422 |
| AC | HCM | F | 0.6 | 134303 | 216 | 55 |
| AC | HCS | M | 5.4 | 35281 | 257 | 76 |
| AC | HDA | F | 0.3 | 10290 | 133 | 89 |
| AC | HDG | M | 2.7 | 90212 | 2776 | 1042 |
| AC | HDR | F | 2.7 | 44265 | 1252 | 471 |
| AC | HDS | F | 2.5 | 18010 | 1775 | 952 |
| AC | HES | F | 17.7 | 31271 | 3574 | 1981 |
| AC | HEZ | F | 3.4 | 44252 | 3753 | 1935 |
| AC | HFE | F | 6.5 | 12767 | 1298 | 702 |
| AC | HFG | F | 1.6 | 44436 | 940 | 412 |
| AC | LFP | F | 0.6 | 59035 | 343 | 242 |
| ATLL | AN | M | 24.3 | 28545 | 2324 | 45 |
| ATLL | C3 | F | 64 | 15560 | 2969 | 5 |
| ATLL | C4 | F | 20.4 | 64408 | 10315 | 30 |
| ATLL | HCG-LEY | M | 8 | 71393 | 3312 | 2039 |
| ATLL | HDM-LFK | F | 6.3 | 38787 | 2513 | 637 |
| ATLL | JH | F | 31.2 | 76432 | 14639 | 107 |
| ATLL | KD5 | F | 21.2 | 39703 | 2065 | 10 |
| ATLL | LEP | M | 21.2 | 78233 | 10063 | 276 |
| ATLL | LER | M | 52.3 | 7017 | 1053 | 7 |
| ATLL | LEU | F | 45.5 | 19440 | 6656 | 6 |
| ATLL | LEZ | M | 26.7 | 86061 | 2843 | 243 |
| ATLL | LFA | F | 10.6 | 30600 | 4293 | 1025 |
| ATLL | LFC | F | 12.3 | 29217 | 5661 | 2426 |
| ATLL | LFE | F | 7.4 | 28028 | 3768 | 1258 |
| ATLL | P7 | F | 18.3 | 39283 | 12980 | 3182 |
| ATLL | S1 | F | 18.1 | 27415 | 9138 | 829 |
| ATLL | S2 | F | 10.8 | 7531 | 1338 | 234 |
| ATLL | S4 | M | 16.7 | 14621 | 1982 | 629 |
| ATLL | S5 | F | 3.6 | 24410 | 4391 | 53 |
| ATLL | S6 | M | 15 | 24823 | 5880 | 282 |
| HAM-TSP | TAA | F | 2.2 | 70585 | 5995 | 1805 |
| HAM-TSP | TAL | F | 8.7 | 106366 | 12839 | 3024 |
| HAM-TSP | TAN | F | 4 | 21024 | 2083 | 1388 |
| HAM-TSP | TAS | F | 3 | 13485 | 1478 | 901 |
| HAM-TSP | TAT | F | 9.6 | 10525 | 2204 | 1281 |
| HAM-TSP | TAW | F | 1.4 | 94529 | 3528 | 1006 |
| HAM-TSP | TAY | F | 1.4 | 66941 | 3582 | 1069 |
| HAM-TSP | TAZ | M | 12.5 | 13636 | 3545 | 1965 |
| HAM-TSP | TBA | F | 9.6 | 27784 | 2679 | 1215 |
| HAM-TSP | TBC | F | 20 | 84806 | 26108 | 7460 |
| HAM-TSP | TBG | F | 14.6 | 76744 | 16617 | 3609 |
| HAM-TSP | TBJ | F | 15.8 | 16907 | 2791 | 1442 |
| HAM-TSP | TBO | F | 7 | 13519 | 1904 | 1066 |
| HAM-TSP | TBP | M | 3.5 | 36279 | 5855 | 3604 |
| HAM-TSP | TBR | F | 4.7 | 47148 | 2949 | 1461 |
| HAM-TSP | TBS | F | 10.4 | 21127 | 5216 | 2396 |
| HAM-TSP | TBU | F | 1.1 | 70258 | 1846 | 749 |
| HAM-TSP | TBW | M | 7.3 | 124980 | 13240 | 3136 |
| HAM-TSP | TCG | F | 3.6 | 28031 | 1678 | 766 |
| HAM-TSP | TCO | F | 1.8 | 17903 | 789 | 497 |
| HAM-TSP | TCQ | M | 5 | 44629 | 3830 | 2155 |
| HAM-TSP | TCR | M | 5.9 | 38437 | 3606 | 2048 |
| HAM-TSP | TCT | F | 5.5 | 26719 | 3407 | 1943 |
| HAM-TSP | TDA | M | 6.2 | 7125 | 1165 | 805 |
| HAM-TSP | TW | F | 8.9 | 112958 | 16420 | 3749 |

PVL : proviral load – number of copies per 100 cells; AC: Asymptomatic Carriers; ATLL: Adult T-cell Leukaemia/Lymphoma; HAM/TSP: HTLV-1-Associated Myelopathy / Tropical Spastic Paraparesis.

**Additional references:**

1. Gillet NA, Malani N, Melamed A, Gormley N, Carter R, et al. (2011) The host genomic environment of the provirus determines the abundance of HTLV-1-infected T-cell clones. Blood 117: 3113-3122.
