# Supplementary material for: Genome-wide Determinants of Proviral Targeting, Clonal Abundance and Expression in Natural HTLV-1 Infection
Source: PLoS Pathog. 2013 Mar 21;9(3):e1003271. doi: 10.1371/journal.ppat.1003271 (PMC3605240; doi:10.1371/journal.ppat.1003271)
Supplement: Table S3 — List of annotations datasets used. (DOC) [file ppat.1003271.s011.doc]

**Table S3: A**nnotations datasets used.

| Annotation | Cell type | reference | peak calling algorithm | Max. OR |
| --- | --- | --- | --- | --- |
| RefSeq genes | N/A | [1] | N/A |  |
| CpG islands |  |  |  |  |
| NRSF(REST) | Jurkat | [2,3]; | SISSRs | 167.57 |
| CTCF | pr CD4 | [2,4] | SISSRs | 12.89 |
| BRG1 | HeLa | [5] | Peak-Seq | 2.31 |
| Ini1 |  |  |  | 2.75 |
| BAF155 |  |  |  | 2.62 |
| BAF170 |  |  |  | 1.81 |
| cJun | K562 | [6] | Peak-Seq | 2.33 |
| cFos | GM12878 | [7] | SISSRs | 39.15 |
| cMyc |  |  |  | 8.25 |
| JunD |  |  |  | 30.49 |
| E2f4 | GM06990 | [8] | See ref | 2.99 |
| E2f6 | K562 | [9] | SISSRs | 7.45 |
| Foxp3 | Act. CD4 | [10] | MACS | 5.21 |
|  | Treg |  |  | 6.98 |
| GATA1 | K562 | [11] | SISSRs | 19.21 |
| GATA2 |  |  |  | 17.79 |
| NFkB | GM12878 | [12] | Peak-Seq | 2.62 |
| STAT1 | Act. CD4 | [13] | SISSRs | 335.45 |
| STAT1IFN | IFNγ Stim. CD4 |  |  | 8.18 |
| Yy1 | K562 | Myers lab for the ENCODE project | MACS | 2.28 |
| Znf263 | K562 | [14] | Sole-Search | 3.66 |
| CBP | pr CD4 | [15] | SISSRs | 11.43 |
| p300 |  |  |  | 48.88 |
| MOF |  |  |  | 31.93 |
| Tip60 |  |  |  | 33.45 |
| PCAF |  |  |  | 34.90 |
| HDAC1 |  |  |  | 14.30 |
| HDAC2 |  |  |  | 93.15 |
| HDAC3 |  |  |  | 223.49 |
| HDAC6 |  |  |  | 91.80 |
| p53 | IMR90 | [16] | SISSRs | 343.70 |
| Rad21 | GM12878 | Myers lab for the ENCODE project | MACS | 1.64 |
| SUZ12 (PRC2) | K562 | [17] | See ref | 3.76 |

Act. Activated; Stim. Stimulated; Max. maximum

**Additional references:**

1. Fujita PA, Rhead B, Zweig AS, Hinrichs AS, Karolchik D, et al. (2011) The UCSC Genome Browser database: update 2011. Nucleic Acids Res 39: D876-882.

2. Jothi R, Cuddapah S, Barski A, Cui K, Zhao K (2008) Genome-wide identification of in vivo protein-DNA binding sites from ChIP-Seq data. Nucleic Acids Res 36: 5221-5231.

3. Johnson DS, Mortazavi A, Myers RM, Wold B (2007) Genome-wide mapping of in vivo protein-DNA interactions. Science 316: 1497-1502.

4. Barski A, Cuddapah S, Cui K, Roh TY, Schones DE, et al. (2007) High-resolution profiling of histone methylations in the human genome. Cell 129: 823-837.

5. Euskirchen GM, Auerbach RK, Davidov E, Gianoulis TA, Zhong G, et al. (2011) Diverse roles and interactions of the SWI/SNF chromatin remodeling complex revealed using global approaches. PLoS Genet 7: e1002008.

6. Raha D, Wang Z, Moqtaderi Z, Wu L, Zhong G, et al. (2010) Close association of RNA polymerase II and many transcription factors with Pol III genes. Proc Natl Acad Sci U S A 107: 3639-3644.

7. Rozowsky J, Abyzov A, Wang J, Alves P, Raha D, et al. (2011) AlleleSeq: analysis of allele-specific expression and binding in a network framework. Mol Syst Biol 7: 522.

8. Lee BK, Bhinge AA, Iyer VR (2011) Wide-ranging functions of E2F4 in transcriptional activation and repression revealed by genome-wide analysis. Nucleic Acids Res 39: 3558-3573.

9. Trojer P, Cao AR, Gao Z, Li Y, Zhang J, et al. (2011) L3MBTL2 protein acts in concert with PcG protein-mediated monoubiquitination of H2A to establish a repressive chromatin structure. Mol Cell 42: 438-450.

10. Birzele F, Fauti T, Stahl H, Lenter MC, Simon E, et al. (2011) Next-generation insights into regulatory T cells: expression profiling and FoxP3 occupancy in Human. Nucleic Acids Res 39: 7946-7960.

11. Fujiwara T, O'Geen H, Keles S, Blahnik K, Linnemann AK, et al. (2009) Discovering hematopoietic mechanisms through genome-wide analysis of GATA factor chromatin occupancy. Mol Cell 36: 667-681.

12. Kasowski M, Grubert F, Heffelfinger C, Hariharan M, Asabere A, et al. (2010) Variation in transcription factor binding among humans. Science 328: 232-235.

13. Liao W, Lin JX, Wang L, Li P, Leonard WJ (2011) Modulation of cytokine receptors by IL-2 broadly regulates differentiation into helper T cell lineages. Nat Immunol 12: 551-559.

14. Frietze S, Lan X, Jin VX, Farnham PJ (2010) Genomic targets of the KRAB and SCAN domain-containing zinc finger protein 263. J Biol Chem 285: 1393-1403.

15. Wang Z, Zang C, Cui K, Schones DE, Barski A, et al. (2009) Genome-wide mapping of HATs and HDACs reveals distinct functions in active and inactive genes. Cell 138: 1019-1031.

16. Botcheva K, McCorkle SR, McCombie WR, Dunn JJ, Anderson CW (2011) Distinct p53 genomic binding patterns in normal and cancer-derived human cells. Cell Cycle 10: 4237-4249.

17. Ram O, Goren A, Amit I, Shoresh N, Yosef N, et al. (2011) Combinatorial patterning of chromatin regulators uncovered by genome-wide location analysis in human cells. Cell 147: 1628-1639.
