# Supplementary material for: Genome-wide Determinants of Proviral Targeting, Clonal Abundance and Expression in Natural HTLV-1 Infection
Source: PLoS Pathog. 2013 Mar 21;9(3):e1003271. doi: 10.1371/journal.ppat.1003271 (PMC3605240; doi:10.1371/journal.ppat.1003271)
Supplement: Table S6 — Tax sorting experiment – sample data. (DOC) [file ppat.1003271.s014.doc]

**Table S6: Tax sorting experiment – sample data**

| Patient # | CD4+Tax+ cells | CD4+Tax+ /CD4+ | CD4+Tax+ /total | CD4+Tax- cells | CD4+Tax-/CD4+ | CD4+Tax- /total |
| --- | --- | --- | --- | --- | --- | --- |
| 1 | 497,369 | 38.1% | 7.0% | 781,154 | 60.0% | 11.1% |
| 2 | 644,048 | 41.6% | 10.8% | 893,925 | 56.1% | 14.5% |
| 3 | 136,793 | 29.2% | 9.8% | 326,983 | 69.8% | 23.4% |
| 4 | 461,568 | 21.5% | 9.0% | 1,866,092 | 77.9% | 32.6% |
| 5 | 242,228 | 10.8% | 3.8% | 2,368,791 | 88.4% | 31.1% |
| 6 | 242,984 | 11.9% | 6.3% | 1,957,727 | 87.3% | 46.2% |
| 7 | 106,896 | 16.4% | 3.9% | 583,892 | 81.8% | 19.3% |
| 8 | 222,177 | 19.4% | 3.1% | 943,268 | 79.1% | 12.5% |
| 9 | 165,110 | 23.6% | 5.6% | 587,502 | 73.1% | 17.2% |
| 10 | 925,785 | 53.8% | 23.2% | 578,717 | 32.8% | 14.2% |
| Total cells | 3,644,958 |  |  | 10,888,051 |  |  |
| Total DNA (μg) | 4.067 |  |  | 8.19 |  |  |
| Total proviral copies found | 20813 |  |  | 10326 |  |  |
